# Expression Analysis of *PIN* Genes in Root Tips and Nodules of *Lotus japonicus*

**DOI:** 10.3390/ijms20020235

**Published:** 2019-01-09

**Authors:** Izabela Sańko-Sawczenko, Dominika Dmitruk, Barbara Łotocka, Elżbieta Różańska, Weronika Czarnocka

**Affiliations:** Department of Botany, Faculty of Agriculture and Biology, Warsaw University of Life Sciences, Nowoursynowska 159, 02-776 Warsaw, Poland; izabela_sanko_sawczenko@sggw.pl (I.S.-S.); dominika_dmitruk@sggw.pl (D.D.); barbara_lotocka@sggw.pl (B.Ł.); elzbieta_rozanska@sggw.pl (E.R.)

**Keywords:** *Lotus japonicus*, root nodule, PIN-protein, auxin

## Abstract

Auxins are postulated to be one of the pivotal factors in nodulation. However, their transporters in *Lotus japonicus*, the model species for the study of the development of determinate-type root nodules, have been scarcely described so far, and thus their role in nodulation has remained unknown. Our research is the first focusing on polar auxin transporters in *L. japonicus*. We analyzed and compared expression of *PINs* in 20 days post rhizobial inoculation (dpi) and 54 dpi root nodules of *L. japonicus* by real-time quantitative polymerase chain reaction (qPCR) along with the histochemical β-glucuronidase (*GUS*) reporter gene assay in transgenic hairy roots. The results indicate that *LjPINs* are essential during root nodule development since they are predominantly expressed in the primordia and young, developing nodules. However, along with differentiation, expression levels of several *PINs* decreased and occurred particularly in the nodule vascular bundles, especially in connection with the root’s stele. Moreover, our study demonstrated the importance of both polar auxin transport and auxin intracellular homeostasis during *L. japonicus* root nodule development and differentiation.

## 1. Introduction

Nitrogen is a crucial component in the biosynthesis of amino and nucleic acids, which makes it a pivotal nutrient for plants. Due to its restricted availability in soil, it is also one of the most common limiting factors of plant growth. In spite of its high content in the atmosphere, plants are not adapted to assimilate it directly in its dinitrogen form. Symbiosis with nitrogen-fixing bacteria—rhizobia—allows fabaceans to exploit the nitrogen-rich atmospheric source. This kind of relationship of the plants with rhizobia manifests itself in the formation of root nodules and is an evolutionary achievement of fabacean plants. As a result of the symbiosis, rhizobia obtain photosynthesis-derived carbon compounds from the host while host plants benefit by gaining access to the dinitrogen reduced by rhizobia into ammonia, which is suitable for utilization by plants [1,2].

*Lotus japonicus* is a fabacean model species, forming determinate-type root nodules. These kinds of nodules are usually spherical and are characterized by a lack of persistent meristem or developmental zonation in their anatomy [1]. The microsymbiont, *Mesorhizobium loti*, invades *L. japonicus* through an infection thread originating from a deformed root hair. At this stage, a nodule primordium with determinate meristem starts to develop in the root cortex beyond the site of infection. Rhizobia migrate in the infection thread to the infection droplet and then, enclosed in a host-derived peribacteroid membrane, are released into the host cell as symbiosomes [3]. Inside the symbiosome, bacteria differentiate into their dinitrogen-fixing form—bacteroids [4]. In the *L. japonicus* root nodules, it has been shown that the morphology of bacteroids is not very different from that observed for free-living rhizobia [3]. Infected cells in the determinate-type of nodules differentiate synchronously, which leads to the formation of a homogenous population of bacteroids [5]. The mature *L. japonicus* nodule consists of a central, bacteroid-containing, parenchymatous tissue surrounded by the nodule cortex with a vascular system [3].

Auxins are plant hormones, which are synthesized in the apical tissues and demonstrate the ability to be accumulated in distant organs. They are involved in various physiological processes, such as embryogenesis, organogenesis, meristem activity maintenance, tropisms, differentiation of vascular tissues, root elongation, apical dominance, fruit ripening and growth responses to many environmental stimuli [6]. Auxins can be transported by two divergent tracks: through phloem parenchyma—fast, non-polar transport—or by cell-to-cell polar auxin transport (PAT). The second track is much slower and requires special membrane transporters, but is essential for local auxin accumulation. The protonated form of indole-3-acetic acid (IAA), IAAH, is hydrophobic and enters the cell passively through the plasma membrane. Because the pH in the cytoplasm is slightly alkaline, the proton dissociates from the IAA molecules. The deprotonated IAA^−^ cannot passively diffuse out from the cell, but needs to be transported actively by specific auxin efflux carriers—principally PIN-formed proteins (PINs). Their asymmetrical (polar) distribution within the cell membrane determines the direction of auxin flow [6]. The subcellular location of PINs is mainly referred to the plasma membrane; however, some *Arabidopsis thaliana* PINs are also located in the endoplasmic reticulum (ER) membranes and mediate auxin flux from the cytosol to the ER lumen. These particular PIN proteins, which are AtPIN5, AtPIN6 and AtPIN8, form the so-called PIN5-like subclade [7,8,9].

Auxins, as well as PINs, are postulated to play an essential role in the development and meristematic activity maintenance of indeterminate-type root nodules [10,11,12,13,14]. For example, in *Trifolium repens* auxin accumulation in developing root nodules is preceded by local inhibition of PAT [15]. In *Medicago truncatula*, on the other hand, treatment with auxin transport inhibitors resulted in pseudonodule formation [12]. Additionally, there are strong indications that PAT along with the main auxin transporters play an essential role in the development [11] and meristematic activity maintenance [13] of *M. truncatula* root nodules. Also, it was previously found that during early stages of *M. truncatula* nodule formation, cytokinin signaling leading to flavonoid accumulation is required for local changes in PAT and subsequent auxin accumulation in cortical cells [16]. Moreover, there are evidences, which support the idea that the indeterminate and determinate types of root nodules have different auxin requirements. For example, inoculation with IAA-overproducing rhizobia enhanced nodulation in *M. truncatula*, whereas it had no impact on nodulation in *Phaseolus vulgaris* bearing determinate-type root nodules [17]. Different auxin requirements, depending on the nodule type, were also indicated for *L. japonicus* and *Glycine max* in comparison, again, to *M. truncatula* [18,19,20,21].

The development of *L. japonicus* determinate-type root nodules seems to imply that auxins are principally operational in triggering proliferation of cortical cells as well as the development of cortical lenticels and vascular bundles [22,23,24,25,26]. Although LjPIN proteins have been recently identified [27], their putative involvement in *L. japonicus* root nodule development has not yet been investigated. Therefore, this study is the first attempt to explore the possible contribution of polar auxin transporters—PIN proteins—in the morphogenesis of the *L. japonicus* determinate root nodules. Except for the bioinformatic analysis of the LjPIN sequences, our study provides a detailed investigation of their expression during root nodule development.

## 2. Results

### 2.1. Bioinformatic Analysis of L. japonicus PIN Sequences Reveals Significant Similarity to Their M. truncatula and A. thaliana Orthologs and a Typical Transmembrane Topology

We performed a detailed analysis of LjPIN gene/protein sequences. Our finding mostly, but not entirely, coincides with the study by Kohlen et al. 2017 [27]. By comparing previously identified PIN protein sequences of *A. thaliana* and *M. truncatula* with the Kazusa database [28,29], eight functional members of the PIN transporter family were found in *L. japonicus* (Table 1). Sequences assigned as Lj4g3v3114900.1 and Lj2g3v0661480.1 were treated as a single locus encoding LjPIN1 since they were identical. In order to resolve the direct orthology among these three model species, both protein and coding sequences of *L. japonicus* PINs were compared to *A. thaliana* and *M. truncatula* protein and nucleotide collections, respectively. Based on the results, direct orthologs were matched (Table 1). However, it should be noted that for AtPIN3, AtPIN4 and AtPIN7 a more appropriate approach is to describe them together, as one, closely related subfamily of paralogs, as previously indicated [30].

Although the study by Kohlen et al. 2017 claimed the existence of two distinct orthologs of AtPIN8 in the *L. japonicus* genome, the sequence Lj3g3v3735560—assigned to LjPIN8 [27]—seems to be partial or inaccurately sequenced and annotated in the database, hence, it has not been taken into consideration in our study. However, the existence of other, unidentified PIN proteins in the *L. japonicus* cannot be absolutely ruled out until the whole genome is completely assembled and carefully analyzed. In this study, we propose Lj2g3v1034600.1 as the only ortholog of AtPIN8 and designate it as LjPIN8. According to the sequence analysis, LjPIN8 along with LjPIN5 and LjPIN6 are the orthologs of *A. thaliana* PIN5-like subclade members. PIN proteins from this subclade are predominantly targeted to the ER [7,8,9], contrary to other PINs, which are exclusively located in the plasma membrane [31]. A more detailed phylogenetic relationship among PIN proteins is described in the cladogram (Figure S1).

We also investigated the transmembrane topology of all identified LjPINs using the TMHMM tool [32]. We found conserved transmembrane helices in both C- and N-termini of the seven LjPINs and a hydrophilic loop between them (Figure S2), which is consistent with the typical structure of PIN-formed family proteins [31]. There was an assumption that one of the PIN proteins—LjPIN6—is incorrectly assigned in the Kazusa database [27] with two different accession numbers: Lj0g3v0178829 (LjPIN6a, probable N-terminus) and Lj1g3v0264160.1 (LjPIN6b, possible C-terminus) [27]. Our quantitative polymerase chain reaction (qPCR) analysis revealed that their expression level in *L. japonicus* root nodules appeared to be identical (Figure S3). Thus, to examine whether LjPIN6b is situated on the same chromosome, downstream to LjPIN6a, and whether both sequences could constitute one gene, we designed a pair of primers, from which the forward one was anchored to the end fragment of LjPIN6a, while the reverse one to the beginning of the LjPIN6b coding sequence. The performed RT-PCR analysis proved that LjPIN6a and LjPIN6b, in fact, constituted one sequence since they were jointly transcribed (Figure S4). Nevertheless, the complete amplicon was longer than expected, thus the PCR product was sequenced, and the full-length LjPIN6 sequence is provided (Figure S5A). The transmembrane domain prediction using the complete, assembled sequence of LjPIN6 was repeated. This allowed us to identify transmembrane domains, analogous to those from *A. thaliana* and *M. truncatula* PIN6 orthologs, at both N- and C-termini. The positions, lengths, and number of domains were similar to those of *MtPIN6* and *AtPIN6* (Figure S5B).

### 2.2. Real-Time Quantitative Polymerase Chain Reaction (qPCR) Expression Analysis of LjPINs Reveals Differences among Root Tips and Root Nodules at Both Tested Developmental Stages

In order to investigate whether any of the eight *LjPINs* may play an important role in root nodule development, we performed an analysis of their expression in young nodules harvested 20 days post rhizobial inoculation (dpi), and in mature 54 dpi nodules. Additionally, root tips collected from plants 20 dpi were tested as a control, to obtain a better picture of *LjPINs* expression pattern within root tissues.

**Figure 1:**
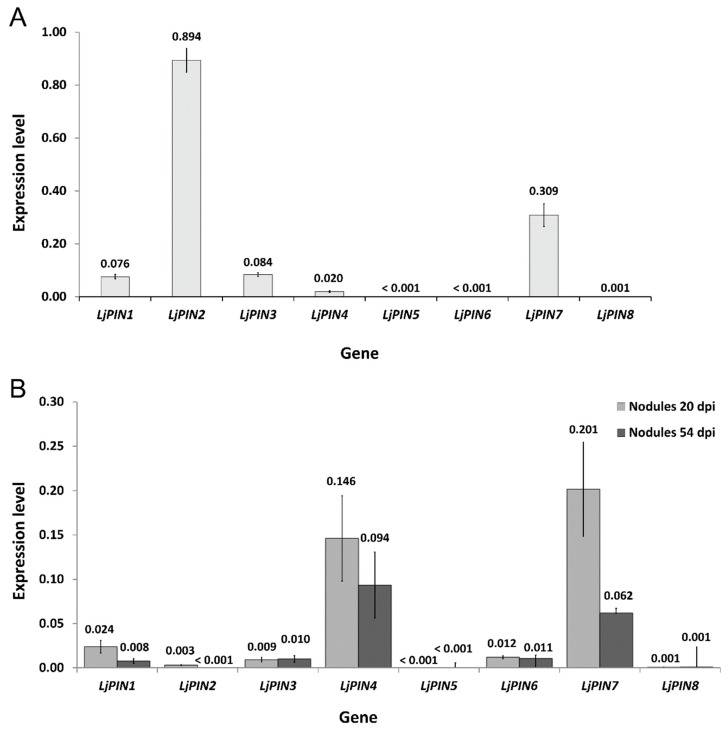
Normalized expression level of *LjPINs* in root tips (**A**) and nodules (**B**). Mean values (±standard error (SE)) are derived from three biological replicates, for which three individual quantitative polymerase chain reactions (qPCR) were performed (*n* = 9).

It was confirmed that all tested *LjPINs* were expressed in the root tips and in both analyzed developmental stages of root nodules. However, the expression of *LjPIN5* and *LjPIN8* was generally marginal (Figure 1A,B). In the root tips, the highest absolute level of expression among all *LjPINs* was attributed to *LjPIN2*, whereas there was very low transcript abundance of *LjPIN5*, *LjPIN6* and *LjPIN8* (Figure 1A). In young, developing nodules, *LjPIN1*, *LjPIN4* and *LjPIN7* demonstrated the highest expression levels, while the transcript levels of *LjPIN5* and *LjPIN8* were insignificant (Figure 1B).

**Figure 2:**
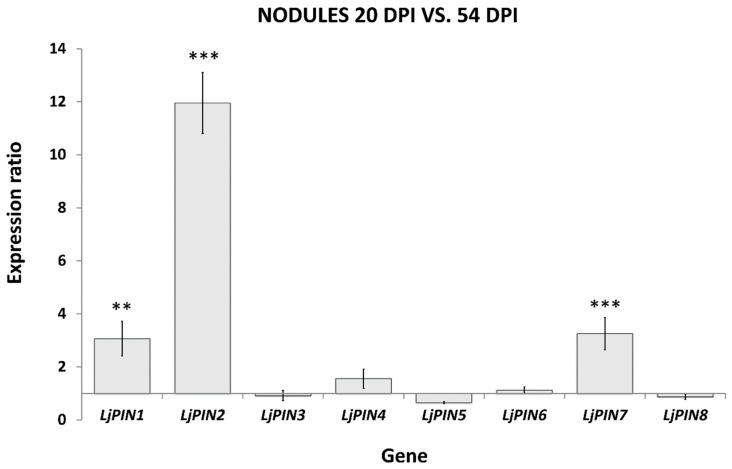
Relative expression levels of *LjPINs* in root nodules 20 dpi in comparison to the nodules 54 dpi. The expression values for nodules 54 days post rhizobial inoculation (dpi) were set as unity for each tested gene and the expression level in nodules 20 dpi was proportionally converted. Mean values (±SE) are derived from three biological replicates, for which three individual qPCR reactions were performed (*n* = 9). Asterisks above the bars represent statistically significant difference at the level *p* < 0.01 (**) or *p* < 0.001 (***).

The comparison of PIN expressions in two tested developmental stages of root nodules demonstrated that *LjPIN1*, *LjPIN2* and *LjPIN7* were significantly upregulated in the 20 dpi root nodules compared to the mature ones, harvested 54 dpi (Figure 2). Statistical analysis revealed that *LjPIN3*, *LjPIN4*, *LjPIN5, LjPIN6* and *LjPIN8* were the *LjPINs* with unchanged expression levels during nodule maturation (Figure 2). Since the expression levels of *LjPIN5* and *LjPIN8* were very low in each of the samples studied, we performed PCR on a genomic DNA template to confirm whether the primers for qPCR amplification were properly designed. In both cases, we received products of the expected length (Figure S6).

### 2.3. Spatial Expression Patterns of PIN Genes Strongly Indicate Their Essential Role in the Development of L. Japonicus Root Nodules and in the Formation of the Polar Auxin Transport Pattern in the Root Tips

**Figure 3:**
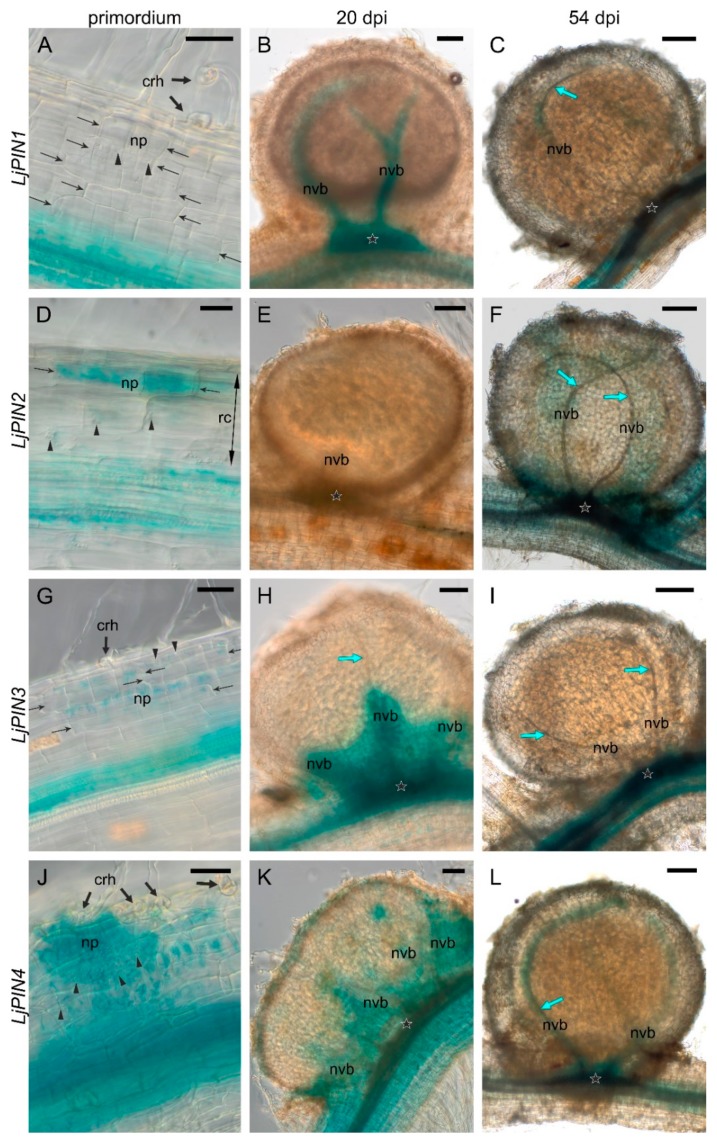
Expression patterns of *LjPIN1*, *LjPIN2*, *LjPIN3*, and *LjPIN4* in nodule primordium (**A**,**D**,**G** and **J**—respectively), 20 dpi nodule (**B**,**E**,**H** and **K**—respectively) and mature, 54 dpi nodule (**C**,**F**,**I** and **L**—respectively), obtained by GUS histochemical assays performed on transgenic roots. np—nodule primordium; rc—root cortex; crh—curled root hair; nvb—nodule’s vascular bundle; black thin arrows—cells of root cortex in the division; arrowheads—starch grains; blue arrows—tracheal elements; stars—vascular connection between root’s stele and nodule. Bars: 20 µm (**D**), 50 µm (**A**,**G**,**J**), 100 µm (**B**,**E**,**H**,**K**), 200 µm (**C**,**F**,**I**,**L**).

**Figure 4:**
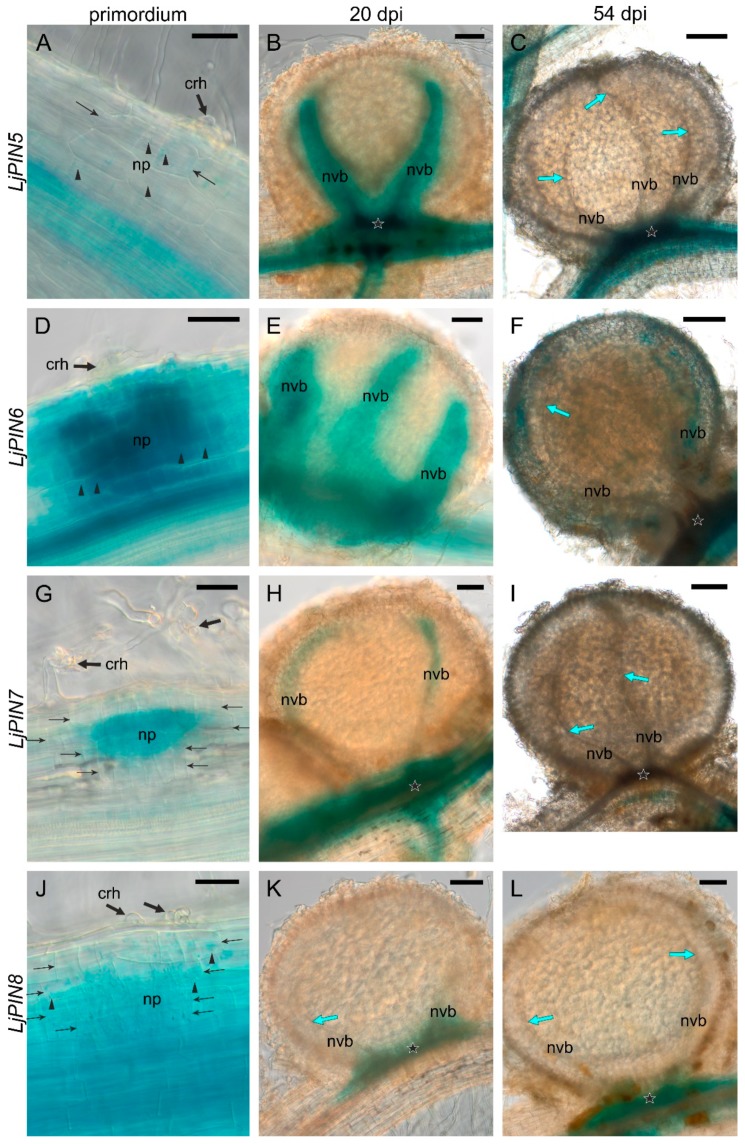
Expression patterns of *LjPIN5*, *LjPIN6*, *LjPIN7*, and *LjPIN8* in nodule primordium (**A**,**D**,**G** and **J**—respectively), 20 dpi nodule (**B**,**E**,**H** and **K**—respectively) and mature, 54 dpi nodule (**C**,**F**,**I**, **L**—respectively), obtained by GUS histochemical assays performed on transgenic roots. np—nodule primordium; crh—curled root hair; nvb—nodule’s vascular bundle; black thin arrows—cells of root cortex in the division; arrowheads—starch; blue arrows—tracheal elements; stars—vascular connection between root’s stele and nodule. Bars: 50 µm (**A**,**D**,**G**,**J**), 100 µm (**B**,**E**,**H**,**K**,**L**), 200 µm (**C**,**F**,**I**).

In order to evaluate the expression of *LjPINs* in root and nodule tissues, we performed transient transformation of *L. japonicus* roots with genetic constructs bearing each of the *LjPINs*’ promoters (*pLjPIN*) fused with the β-glucuronidase (*GUS*) gene, and subsequent histochemical localization of GUS activity. Twenty days after inoculation with rhizobia, transgenic hairy roots showed a broad spectrum of nodule development stages, from the stage of the first cell divisions in the outer layers of the primary root cortex (initial primordia), through the stages of the nodule primordium still hidden in the root cortex (the “hidden” primordia), stage of primordia exposed over the root surface (the “emerged” primordia), finally to the formation of nodules with cortex, vascular bundles and evident bacteroid tissue (20-day-old or close to this age). Moreover, developmentally inactive early primordia (abortive) were found occasionally, characterized by a lack of starch in the region of divided root cortex cells. In the roots sampled at 54 dpi, in addition to all the younger stages, mature nodules (i.e., with the senescence probably initiated) were present, but nodules with symptoms of advanced degradation of bacteroid-containing tissue were not yet observed (Figure 3 and Figure 4).

The transgenic roots were sufficiently thin to image the tissues throughout their whole thickness, which was useful in the search for initial nodule primordia. The young and mature nodules had a larger diameter and lower transparency. Nevertheless, images of tracheary elements within the nodule vascular bundles and the images of their vascular connection with the stele were obtained, which was sufficient to determine the histological location of the signal. The nodule expression patterns of all *LjPIN* genes are summarized in Table 2.

During the root nodule development, the expression pattern of the ***LjPIN1*** underwent changes. In the initial primordia, i.e., primordia composed of a few to over a dozen primary cortex cells, a signal was absent (Figure 3A). In the “emerged” primordia, strong signals appeared within their base, i.e., at the junction of the nodule vasculature and root stele, as well as in the differentiating nodule vascular bundles together with the adjacent nodule cortex (Figure S7B). In young nodules at 20 dpi, *LjPIN1* expression persisted within their base and in vascular bundles (Figure 3B), while in the mature nodules at 54 dpi, only weak signal was present in some sectors of vascular bundles (Figure 3C).

The expression of ***LjPIN2*** was present in the initial primordia, and it was strongest in their rhizodermis-facing part (Figure 3D). A strong signal was observed throughout the entire older primordia, as well as in the root cortex cells adjacent to them (Figure S8A). In the “emerged” primordia, the strongest signal was observed in the nodule cortex, as well as in the differentiating vascular bundles and their connection to the root stele (Figure S8C). No signal of *LjPIN2* was observed in nodules at 20 dpi (Figure 3E). However, in the nodules formed at 54 dpi, *LjPIN2* was weakly expressed in some sectors of vascular bundles and in the nodule base (Figure 3F).

In the initial primordia, the ***LjPIN3*** signal was present uniformly in divided root cortex cells (Figure 3G). In young primordia, signal-positive curled root hairs were occasionally found, and they were interpreted as infections that were “allowed” to develop further by the host plant (Figure S9A). The signal was strongest in the pericycle cells facing the developing primordia. In the “hidden” primordia, *LjPIN3* expression was present in their entire volume (Figure S9B), whereas in the “emerged” primordia, it was limited to two places: the connection with the root stele, and the primordium apex, in the vicinity of the infection site (base of the root hair cell and adjacent primordium cells) (Figure S9C). In nodules at 20 dpi, the *LjPIN3* signal usually appeared only in the vascular connection with the root stele, and occasionally in the proximal part of the vascular bundles (Figure 3H). In the nodules formed at 54 dpi, *LjPIN3* expression was not discernible within the nodule, but present in the adjacent root stele (Figure 3I).

***LjPIN4*** was expressed in the divided cells of the root cortex constituting the initial primordia (Figure 3J). The *LjPIN4* signal intensified with the primordium growth (Figure S10C). In the “emerged” primordia, very strong signal was discernible in the cortex, vascular bundles and connection with the root stele (Figure S10D). In the nodules at 20 dpi, the *LjPIN4* was expressed within the vascular connection with the stele and within nodule base (Figure 3K), and the signal extended into vascular bundles, where it disappeared toward the nodule apex. In the nodules at 54 dpi, the *LjPIN4* signal was narrowly associated with vascular bundles and vascular connection with root stele (Figure 3L).

In the initial primordia, the ***LjPIN5*** signal was weak, and its presence correlated positively with the presence of starch (Figure 4A). The “hidden” primordia did not show the *LjPIN5* expression (Figure S11B–E). In the “emerged” primordia, the *LjPIN5* signal was found in their entire volume except for the cortex, and it was stronger in the base of the nodule and in the developing vascular bundles (Figure S11F). The nodules at 20 dpi showed *LjPIN5* expression in the vascular connection with the stele and in the vascular bundles up to their apices (Figure 4B). However, in the nodules 54 dpi, signal persisted only in the vascular connection with the stele (Figure 4C).

The initial primordia of the nodules showed clear ***LjPIN6*** signals in the divided root cortex cells and the signal was strengthened in the pericycle opposite the curled root hair (Figure 4D). Signals were also observed in the curled root hair in the starch-less primordia and vice versa. In nodules at 20 dpi, the *LjPIN6* was expressed in their base and along the vascular bundles to their apex (Figure 4E). A nodule sized as the 20 dpi one was also found in the relatively young root part (with young lateral roots), which had a signal only within its base (Figure S12A). This variability in the *LjPIN6* expression pattern could be attributed to the nodulation autoregulation system action. In the nodules formed at 54 dpi, strong *LjPIN6* signal was visible within the vascular connection with the stele, and a discontinuous one along the vascular bundles (Figure 4F).

In the initial primordia, ***LjPIN7*** was expressed only in the divided cells located centrally in the primordium (Figure 4G). Older primordia were uniformly stained (Figure S13C). In the “emerged” primordia, strong signal appeared along the vascular bundles and in the vascular connection with the root stele (Figure S13D). In the nodules at 20 dpi, *LjPIN7* signal was present in the nodule base, and discontinuously in vascular bundles (Figure 4H), while the nodules at 54 dpi did not exhibit *LjPIN7* expression (Figure 4I).

In the initial primordia, ***LjPIN8*** expression was present in divided cortical cells (Figure 4J). In older primordia, the signal was weak, but it was strengthened in the root pericycle at the primordia base (Figure S14B). In the nodules formed at 20 dpi and 54 dpi (Figure 4K,L, respectively), the signal appeared only in their base.

**Figure 5:**
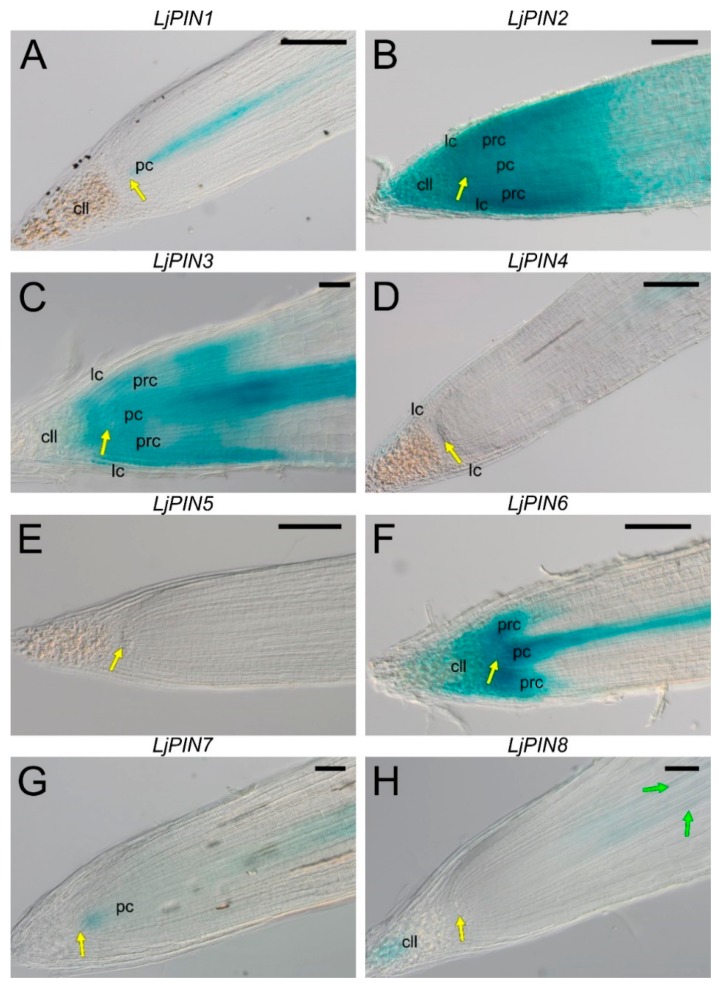
Expression patterns of the *LjPIN1*, *LjPIN2*, *LjPIN3*, *LjPIN4*, *LjPIN5*, *LjPIN6*, *LjPIN7*, and *LjPIN8* (**A**–**H**, respectively), obtained by GUS histochemical assays in representative root tips of transgenic *L. japonicus* plants. pc—procambium; prc—meristematic primary root cortex; cll—columella; lc—lateral root cap; yellow arrows—columella initials-procambial initials boundary; green arrows—distal differentiated tracheary elements. Bars: 20 µm (**A**), 50 µm (**C**,**G**,**H**), 100 µm (**B**,**D**–**F**).

In the root tips, the expression pattern of the *LjPINs* varied, depending on the gene. In root apical meristems (RAMs) of plants containing the *p**LjPIN1**::GUS* construct, the signal was distinct throughout the procambium, including the initial cells (Figure 5A). The expression of ***LjPIN2*** was found throughout the distal part of the root, i.e., the entire RAM and the root cap (Figure 5B). However, in the distal part of the meristematic primary cortex (i.e., in the periblem), the signal was slightly stronger than in other parts of the root apex. Expression of ***LjPIN3*** was detected in the procambium, with the signal being the strongest at the proximal end of the root cap and weakening toward the procambium initials (Figure 5C). Additionally, the expression was visible in the periblem together with the protoderm, as well as in the young layers of the lateral cap and in the nondifferentiated cells of the columella. The expression of the ***LjPIN4*** gene was found in the root stele; however, it disappeared in the elongation zone (Figure 5D) and we did not detect any signal in the RAM. A weak signal was observed in the differentiated cells of the lateral root cap. The root tips of *L. japonicus* did not exhibit any expression of the ***LjPIN5*** gene (Figure 5E). The promoter activity of the ***LjPIN6*** gene was detected in the procambium, columella, lateral cap and periblem (Figure 5F). However, in the latter region of RAM, the signal was weaker, especially in the periblem layers adjacent to the procambium. In the root cap, the signal was disappearing toward its distal end and vanished completely in the differentiated layers of the lateral cap. Expression of the ***LjPIN7*** was weak in the root tips, and occurred only in the region of procambium initial cells and their direct derivatives (Figure 5G), after which it reappeared in the older procambium above the proximal end of the root cap. The ***LjPIN8*** gene was expressed in the distal columella cells (Figure 5H). In addition, a very weak signal was observed in the part of the procambium located distally relative to the youngest differentiated protoxylem elements.

There was no visible signal in the control—non-transformed plants, treated with β-glucuronidase substrate (Figure S15).

### 2.4. Twenty Days Post Inoculation L. japonicus Nodules Still Demonstrate Meristematic Activity

**Figure 6:**
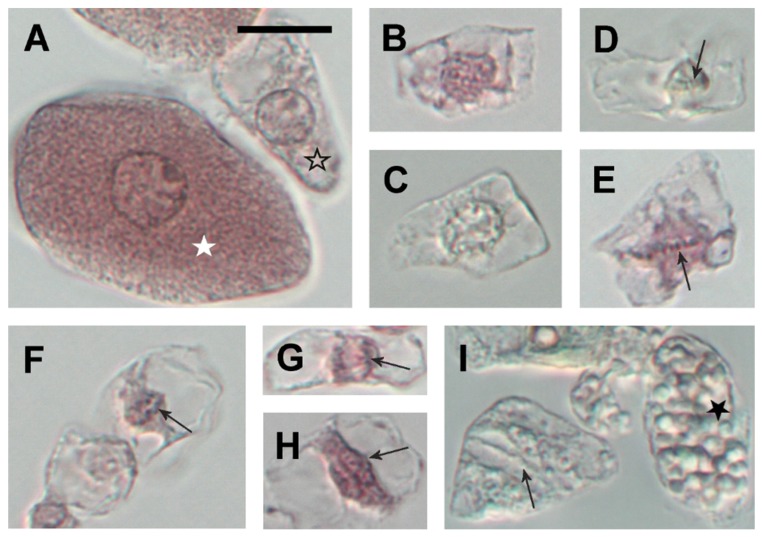
Cell types separated from *L. japonicus* root nodules after maceration in pectinase. Infected cell (white star) and noninfected interphase cell (open star) (**A**). Early and late prophase cells, respectively (**B**,**C**). Metaphase cells (arrows point to the metaphase plate) (**D**,**E**). Anaphase cell (arrow points to one group of chromatids) (**F**). Early and late telophase cell (arrows point to one of the twin nuclei) (**G**,**H**, respectively). Cell in cytokinesis (arrow points to the cell plate) and noninfected fully differentiated cell with large starch grains (black star) (**I**). Images (**A**,**B**,**E**–**H**): cells stained with cold acetocarmine, images (**C**,**D**,**I**): unstained. All images are in the same magnification, bar = 20 µm.

To resolve whether the high expression of *LjPINs* in young, developing nodules could be associated with the residual meristematic activity ongoing in *L. japonicus* nodules, microscopic analysis was performed. It proved the presence of mitotic divisions of noninfected cells in the nodules at 20 dpi (Figure 6). Usually, the dividing cells lacked large starch grains and they were much smaller than nondividing cells. In mature nodules, harvested at 54 dpi, no mitoses were found.

**Figure 7:**
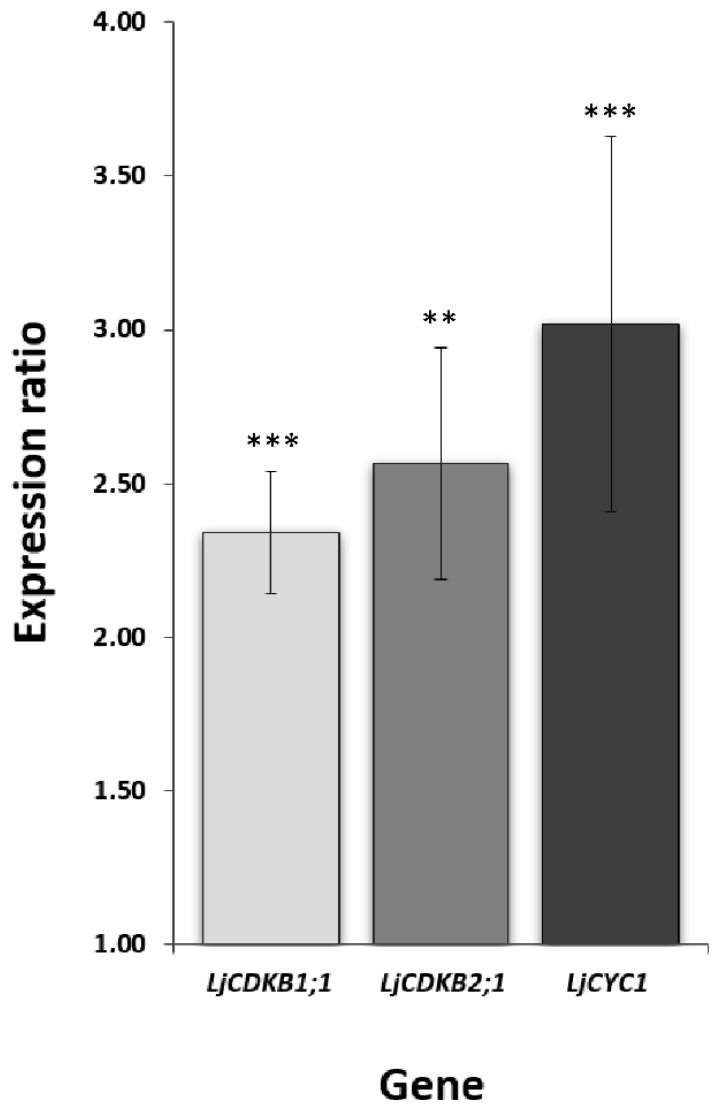
Relative expression level of G2-M phase cell cycle markers. The expression values for root nodules at 54 dpi were set as unity for each tested gene and the expression level in the nodules formed at 20 dpi was proportionally converted. Mean values (±SE) were derived from three biological replicates, for which three individual qPCR reactions were performed (*n* = 9). Asterisks above the bars represent a statistically significant difference in comparison to expression values in the nodules formed 54 dpi, at the level *p* < 0.01 (**) or *p* < 0.001 (***).

To confirm that 20 dpi nodules exhibit mitotic activity, the expression profiles of class B cyclin-dependent kinases (*LjCDKB1;1*, *LjCDKB2;1*) and cyclin 1 (*LjCYC1*) were analyzed. They are the orthologs of *Medicago sativa CDKB1;1* (*cdc2MsD*), *CDKB2;1* (*cdc2MsF*) and *cycMs1*, respectively and have been characterized by their high expression level in the G2 and M phase of the cell cycle [33,34]. Our results of qPCR revealed a significantly higher expression of all *LjCDKB1;1*, *LjCDKB2;1* and *LjCYC1* in young nodules, in comparison to mature ones (Figure 7). Additionally, *LjCDKB2;1* was more highly expressed than *LjCDKB1;1* (Figure S16).

Together, these data indicate that root nodules, harvested 20 dpi, were meristematically active and, therefore, did not complete the differentiation process. In contrast, nodules 54 dpi, which lacked dividing cells and demonstrated a low expression level of *LjCDKB1;1*, *LjCDKB2;1* and *LjCYC1*, were already fully differentiated.

## 3. Discussion

The aim of the present study was to analyze the expression level and spatial expression pattern of *PIN* genes within *L. japonicus* root tissues, especially in the context of the nodulation process. The role of PINs in the root apical meristem has been well described [35] and there is evidence that they are involved in nodulation in fabaceans [11,13,14]. By testing root nodules at different developmental stages, our aim was to select PINs that are potentially crucial for the development of determinate-type root nodules, characteristic for *L. japonicus*.

So far, among *L. japonicus* auxin transporters, only an ATP-binding cassette (ABC) protein—LjABCB1, has been studied within root nodule tissues. It was found to be expressed exclusively in noninfected cells of nodules, adjacent to the infected ones [23].

The present study focused on the distinct auxin transporter family, PIN-formed proteins, which, so far, have been scarcely described. Although a few studies have already identified LjPINs in the *L. japonicus* genome [14,27,36,37], they did not elaborate on their expression nor their potential role in the nodulation. Our study has not only resulted in the characterization of the phylogenetic relation of the PIN proteins to their orthologs from *M. truncatula* and *A. thaliana*, and the prediction of protein transmembrane domains but, most importantly, the investigation of their expression level and spatial expression pattern within root nodules at different developmental stages.

By analyzing the protein sequences of all eight identified LjPINs, it was confirmed that they demonstrate standard transmembrane topology and conserved transmembrane domains in both N- and C-termini with a hydrophilic loop between them. This kind of structure is characteristic of the members of the PIN protein family [31]. We also postulate that the sequence Lj3g3v3735560 originally assigned to *LjPIN8* [27] is probably partial or inaccurately sequenced since there are many overlapping nucleotides (marked as N) in the sequence and it was impossible to define the open reading frame. Therefore, this sequence has not been taken into consideration in our research. Furthermore, our results have confirmed the assumption that sequences Lj0g3v0178829.1 and Lj1g3v0264160.1, in fact, constitute one gene—*LjPIN6* [27]—and are transcribed simultaneously. By sequencing the *LjPIN6* coding DNA sequence (CDS), the gap between the predicted N- and C-terminus of LjPIN6 was determined and its complete sequence is provided.

The analysis of expression of all *LjPINs* by real-time qPCR revealed that *LjPIN2* was admittedly the most highly expressed *PIN* gene in root tips among all identified *LjPINs*. The *A. thaliana* ortholog of LjPIN2 plays an essential role in the optimal gravitropic response in root tips by controlling auxin transport to the root elongation zone [38,39]. Based on the high abundance of the *LjPIN2* transcripts in root tips, it can be assumed that its role is parallel to the *AtPIN2*. Moreover, the spatial expression pattern detected in *L. japonicus* root tips by *GUS* histochemical assay confirms this statement, since the strong signal of *LjPIN2* expression was found in whole root tip, including the RAM and root cap. Comparing spatial expression pattern of *PINs* in *L. japonicus* to the localization of the PIN proteins in the apices of *A*. *thaliana* roots [40,41,42], it can be cautiously assumed that in the root tip of *L. japonicus*, generally more PIN transporters are involved in forming the polar auxin transport pattern than in *A. thaliana*. There are also significant differences in the expression pattern of almost all PIN transporters in the root tips of the mentioned species (Table 3).

The relatively high expression levels of *LjPIN1*, *LjPIN3*, *LjPIN4*, *LjPIN6* and *LjPIN7* in root nodules, harvested at 20 dpi, suggest that at least during differentiation of determinate-type nodules, both polar auxin transport through the plasma membrane and intracellular auxin homeostasis regulation are necessary. *LjPIN1* and *LjPIN7* are both orthologs of *AtPIN1*, richly expressed in *A. thaliana* vascular tissues [43]. Therefore, it can be assumed that the role of *L. japonicus* PIN1 and PIN7 is connected with the development of a nodule’s vascular system. The *A. thaliana* ortholog of LjPIN6, AtPIN6, is one of the PIN-formed proteins belonging to AtPIN5-like subclade localized in the endoplasmic reticulum and suggested to be responsible for auxin transport from the cytoplasm to the ER lumen [7,8,9]. *LjPIN4*, which was abundantly expressed in young root nodules, is most closely related to *AtPIN3*, encoding an auxin transporter, which regulates tropic growth and apical hook development [44,45,46]. Thus, the role of *LjPIN4* in determinate-type root nodule development remains elusive.

We also visualized *pPIN-GUS* expression in transiently transformed *L. japonicus* roots subjected to nodulation. The expression patterns of all the *LjPINs* in the initial primordia, except *LjPIN1*, suggest that the development of nodule primordium requires both generation of PAT, dependent on plasma membrane-bound PIN transporters, and the intracellular regulation of auxin homeostasis, determined by ER-localized PINs. Generally, the expression pattern was most complex in the initial primordia and the “hidden” ones, while in the following stages it underwent gradual simplification and restriction to the nodule base including the vascular connection with the root stele.

Interestingly, the expression of *LjPIN6* was found in the curled root hairs in the starch-less initial primordia, considered as the abortive ones. It was previously proved that the accumulation of auxins in infected root hairs supports the maintenance of infection and the formation of infection threads [47]. Thus, LjPIN6 protein, which is the ortholog of AtPIN6, localized in the ER and involved in the maintenance of subcellular auxin homeostasis [9,48], may participate in the reduction of cytoplasmic auxin concentration, which is unfavorable for the root hair infection and contributes to the early arrest of nodule development.

The spatial expression patterns of *LjPINs* in active nodules at 20 dpi suggests that most of them are involved in vascular bundle development. The exception is *LjPIN2*, which was expressed in initial primordia, but no signal was detected in the nodules 20 dpi. Moreover, the spatial *LjPIN2* expression pattern, determined in this work, coincides with the expression pattern of its ortholog in the indeterminate-type *M. truncatula* root nodules [11]. Furthermore, the expression of *LjPIN8* was detected only in the nodule’s base, which may also explain the marginal level of its expression detected by real-time qPCR, since this site could be easily neglected during nodule detachment for RNA isolation.

In mature nodules formed at 54 dpi, the expression of the majority of *LjPIN* genes disappeared from the differentiated vascular bundles, which is consistent with our real-time qPCR results. Nevertheless, all *LjPINs* were expressed within the nodule base, which suggests the importance of PAT in the functionality of the nodule/stele vascular connection till the end of the nodule life. However, it should be emphasized that the present results do not allow determination of the direction in which the auxin is exported by an individual PIN within various parts of the nodule at the consecutive developmental stages. Therefore, further studies are needed to resolve the details on auxin transport between root stele and nodule.

PIN proteins generate PAT, which is the main cause of a local auxin accumulation. Therefore, it was interesting to compare the pattern of *LjPINs* expression obtained in our study with the data on auxin distribution during the development of *L. japonicus* root nodules. The use of stable *L. japonicus* transformants expressing a *gusA/gfp* fusion gene under control of the soybean auxin-responsive *GH3* promoter [49] revealed the auxin distribution pattern in the nodulating roots [22]. A strong *GUS* signal was found in the dividing outer cortical cells during the nodule primordium formation between 2 and 5 dpi [22]. During primordia development, blue staining was present in the nodule cortex and in the root stele in direct contact with the primordium. In the nodules examined at 21 dpi, *GUS* expression was mainly present in the nodule vascular bundles and occasionally, a weak diffused signal was found in the nodule cortex [22]. Similar results were obtained in work by Takanashi et al. [24], where the same *L. japonicus* GH3::GUS transgenic line was used. Moreover, Suzaki et al. [26] described detailed patterns of auxin accumulation during early nodule development in stable transformants of *L. japonicus* bearing green fluorescent protein (GFP) under the control of a highly active synthetic auxin-responsive promoter *DR5*. In that study, auxin accumulated in pre-primordial cortical cells adjoining curled root hair with infection thread and in the divided cortical cells at the site of infection [26]. A strong GFP signal was maintained until the primordia “emerged” above the root surface. In young nodules formed at 12 dpi, i.e., after differentiation of bacteroid-containing tissue, GFP signal remained in the nodule cortex, including vascular bundles [26]. All those results show a striking spatial coincidence existing between distribution of auxin maxima and *LjPIN* genes expression pattern presented in this work. However, it should be noted that auxin distribution pattern was obtained using a construct bearing a *GH3* promoter from soybean and that four different *GH3* genes are expressed during the development of *L. japonicus* nodules (*LjGH3–3*, *LjGH3–4*, *LjGH3–5* and *LjGH3–18*) [50]. For this reason, the details of actual auxin distribution during nodule differentiation can be more complex.

We assumed that high expression level of several *LjPINs* could be associated with the residual meristematic activity ongoing in the 20 dpi nodules. In order to test this hypothesis, an expression analysis of specific cell cycle markers along with microscopic observations was performed. It proved that at 20 dpi, there was still meristematic activity within nodule tissues in contrast to older, 54 dpi nodules. Moreover, the molecular markers of the G2-M phase [33,34] were significantly upregulated in the young nodules at 20 dpi, compared to the mature, 54 dpi ones. These markers, belonging to class B of cyclin-dependent kinases (CDKB) [51], are highly expressed during the cell cycle and, at the same time, they inhibit endoreduplication in several plant organs [52]. Therefore, by testing the expression levels of their orthologs in *L. japonicus* root nodules, we were convinced that their high expression level was not connected to the endoreduplication cycles in bacteroid-containing cells, but only to the mitotic divisions. All three tested cell cycle markers demonstrated an expression level, which was a few times higher in young, 20 dpi root nodules, compared to their mature, 54 dpi counterparts. Additionally, *LjCDKB2;1* was more highly expressed than *LjCDKB1;1*, which is consistent with the data obtained for their *M. sativa* orthologs [34]. Taken together, these results clearly prove that these two distinct time points corresponded to nodules with active meristem (young, developing) versus those with meristematic activity that was already terminated (differentiated).

Furthermore, microscopic observations of the macerated nodules revealed that in the nodules at 20 dpi, mitoses occurred only in noninfected cells with low starch content, presumably cortical cells. In *L. japonicus*, the accumulation of auxins was demonstrated to occur preferentially in dividing root cortical cells and, after rhizobial colonization of the nodule primordium, it was restricted to the nodule cortical tissues and vascular bundles [22,24,26]. A new model of molecular mechanism assumes that cytokinin signaling in the root cortex, which is activated by infection signals from the rhizodermis, results in auxin accumulation in cortical cells and their successive proliferation, which is obligatory for nodule organogenesis [26]. It was also previously suggested that auxins are required not only for the development of vascular bundles but also for the formation of lenticels on the *L. japonicus* nodule surface [25]. This finding was supported by the study, in which lenticels were not formed on *L. japonicus* nodule surface after treatment with an auxin antagonist, α-(phenyl ethyl-2-one)-indole-3-acetic acid (PEO-IAA) [24].

In conclusion, the present study is the first detailed description of PIN protein sequences and *PIN* genes expression in roots and nodules of *L. japonicus*. On the basis of our results, we postulate that the expression of *PINs* in developing root nodules is connected with auxin accumulation in dividing cortical cells and within the developing vascular system. Moreover, the polar auxin transport seems to be indispensable for maintaining the functionality of the vascular connection between the nodule and the root during the whole nodule life. Furthermore, our results provide strong reasons to consider auxin intracellular homeostasis as an essential factor, which regulates the development and maintenance of determinate root nodules and possibly participates in the autoregulation of nodulation as well. However, to definitely resolve the contribution of each PIN protein in auxin flux during *L. japonicus* root nodule development, phenotypic analysis of PIN mutants together with an intracellular PIN localization study should be performed in the future.

## 4. Materials and Methods

### 4.1. Plant Material, Inoculation with Rhizobia and Growth Conditions

*Lotus japonicus* cv. Gifu B-129 seeds were treated with 96% sulfuric acid (H_2_SO_4_) for 15 min and washed in sterile deionized water five times. Subsequently, seeds were transferred to Petri dishes containing 1% water agar and placed in darkness at 4 °C for 12 h. After this time, the Petri dishes with seeds were placed upside-down in a growth chamber (16 h photoperiod, photosynthetic photon flux density (PPFD) of 80–100 µmol m^−2^ s^−1^ and temperature 25 °C) for four days and then inoculated with *Mesorhizobium loti* strain MAFF303099 culture. Inoculum was prepared by growing bacteria on Tryptone-Yeast medium (0.5% (*w*/*v*) tryptone, 0.3% (*w*/*v*) yeast extract, 6 mM CaCl_2_, pH = 6.8) until the value of culture optical density at 600 nm (OD_600_) was between 0.6 and 0.8. Inoculated *L. japonicus* seedlings were subsequently placed in pots filled with lightweight expanded clay aggregate (LECA) and immediately watered with inoculum diluted 60-fold. Pots were covered with transparent plastic foil for the next 10 days to protect seedlings from drying. After inoculation, plants were grown in constant conditions (16 h photoperiod, PPFD of 110–170 µmol m^−2^ s^−1^ and temperature of 22 °C) for 20 or 54 days before harvest for microscopic analysis and RNA isolation. Plants were watered three times per week: twice with distilled water and once with nitrogen-free Fahraeus medium [53].

### 4.2. Phylogenetic Analysis of LjPINs and Transmembrane Domain Prediction

Sequences of *LjPIN* genes and corresponding proteins were obtained from the Kazusa database for *L. japonicus* v. 3.0 using the BLAST search tool [28,29] by comparing protein sequences of all known *A. thaliana* and *M. truncatula* PINs, retrieved from the UniProt database [54]. Only *L. japonicus* protein sequences with 70–90% identity to known MtPINs were taken into consideration. All found LjPIN sequences were then analyzed by BLAST search within the NCBI database [55]. Full protein sequence alignment to *A. thaliana* and *M. truncatula* proteome was performed using a blastp algorithm, while *LjPINs’* CDS alignment to *A. thaliana* and *M. truncatula* nucleotide collection was completed by discontiguous megablast. The closest orthologs were selected in accordance with the highest MAX score from NCBI BLAST results. Subsequently, the protein sequences of *LjPINs*, MtPINs and AtPINs were used to create phylogenetic trees with the help of the Phylogeny.fr tool [56].

Transmembrane domain prediction was accomplished using a TMHMM Server v. 2.0 [32]. To identify the transmembrane helices within LjPINs, full protein sequences, obtained previously from the Kazusa database v. 3.0 for *L. japonicus* [28,29], were used.

### 4.3. RNA Isolation and cDNA Synthesis

RNA was obtained from *L. japonicus* root tips 20 dpi and root nodules, detached from roots in two different stages of development: 20 and 54 days after rhizobial inoculation. The RNA was isolated from three biological replicates, each consisting of root tips or nodules derived from over 100 or 50 plants, for 20 and 54 dpi, respectively. Total RNA isolation was performed using a GeneMATRIX Universal RNA Purification Kit (EURX, Gdańsk, Poland) with the additional step of on-column DNase I digestion. RNA concentration and purity were tested by the spectrophotometric method with Nanodrop 2000 (Thermo Fisher Scientific, Waltham, MA, USA), while its integrity tested by electrophoretic separation in 1% agarose gel. An equivalent amount of each RNA sample was used for cDNA synthesis with a High Capacity cDNA Reverse Transcription Kit (Thermo Fisher Scientific, Waltham, MA, USA).

### 4.4. Reverse Transcriptase PCR (RT-PCR) and Real-Time qPCR

In order to establish the exact LjPIN6 coding sequence, reverse transcription PCR (RT-PCR) in four biological replicates using Phusion™ High-Fidelity DNA Polymerase (Thermo Fisher Scientific, Waltham, MA, USA) was performed. The pair of primers in which the forward one was anchored to the end fragment of *LjPIN6a* (5′-AATTTCAATGAGACGGAGGTCAC-3′) while the reverse one to the beginning of the *LjPIN6b* coding sequence (5′-CGAAAGTTTTCTTCCCACTACTGT-3′) were designed to amplify the gap between *LjPIN6a* and *LjPIN6b*. Reaction conditions are presented in Table S1. The PCR product was sent for sequencing, and the protein sequence of *LjPIN6* was established by an in-silico translation of the obtained coding sequence using the ExPASy translate tool [57].

Real-time qPCR was conducted in the 7500 Fast Real-Time PCR System (Applied Biosystems, Waltham, MA, USA) using PowerUP SYBR Green Master Mix (Thermo Fisher Scientific, Waltham, MA, USA). Each reaction was performed in three biological replicates and three technical repetitions. Reaction conditions are presented in Table S2. Primers were designed with Primer3 software (Primer3Plus, Free Software Foundation, Inc., Boston, MA, USA) [58] and their sequences are presented in Table S3. The specificity of each primer pair was verified using melting curve analysis. Additionally, the specificity of primers for *LjPIN5* and *LjPIN8* amplification was analyzed by PCR on genomic DNA from *L. japonicus* (250 ng per 20-µL reaction). Reactions were performed using DreamTaq DNA Polymerase (Thermo Fisher Scientific, Waltham, MA, USA) with conditions presented in Table S4. Real-time qPCR efficiency was calculated with the help of the LinRegPCR tool [59]. Statistical analysis of the results, which included the calculation of the relative gene expression levels and the significance of the difference between tested samples, was performed using REST2009 [60]. Results were normalized using two reference genes encoding SERINE/THREONINE PHOSPHATASEs (PP2AA1, Lj2g3v1155670 and PP2AA2, Lj2g3v0742070).

### 4.5. Preparation of Genetic Constructs

The promoter region (*pLjPIN1*—1512 bp, *pLjPIN2*—1998 bp, *pLjPIN3*—1789 bp, *pLjPIN4*—1591 bp, *pLjPIN5*—1980 bp, *pLjPIN6*—1289 bp, *pLjPIN7*—1890 bp, *pLjPIN8*—1800 bp—upstream from the ATG site) of every *LjPIN* gene was amplified using Phusion High-Fidelity DNA Polymerase (Thermo Fisher Scientific, Waltham, MA, USA). The precise locations of promoter sequences are provided in Table S5. For some of the promoters, the obtained sequence was shorter than 2 kb, since it was preceded by the upstream CDS. In the case of *pLjPIN6*, it was not possible to define the exact position on the chromosome, since the sequence is located in the unmapped region of the genome. Therefore, the promoter sequence was obtained through the sequencing of PCR product amplified from the genomic DNA with the following primers: F: AATTCTAAGGTGGGGTGGTTAAAAA and R: CTATGATGAACTTAGCGTCCATCTC). This PCR product contained CDS fragment of *LjPIN6a* and 1289 bp upstream from the ATG codon. The final promoter sequence was obtained through PCR with the primers specified in Table S5. All of the sequences of the primers and condition of the PCRs are presented in Tables S5 and S6, respectively. In the case of *pLjPIN3* and *pLjPIN5*, because of the nonspecific (high abundance of adenines (A)) sequence around the ATG site, there was a necessity to apply two distinct PCRs for promoter amplification. In the first one (PCR I), the reverse primer was anchored in the coding sequence of *LjPIN*, and the product of amplification was a template for the second PCR (PCR II) with primers designed to amplify the final promoter region. In the next step, all amplified promoter sequences were elongated with *att* sequences essential for Gateway cloning. Sequences of the primers and the PCR condition are presented in Tables S5 and S7, respectively. Prepared inserts were incorporated into the pKGWFS7 vector, containing β-glucuronidase (GUS) reporter gene [61] according to the guidelines form the Gateway User Manual [62]. The correctness of the inserts was verified by sequencing.

### 4.6. Transient Transformation of L. japonicus Roots and Histochemical Localization of GUS Activity

Transient transformation of the *L. japonicus* hairy roots was done according to the *Lotus japonicus* Handbook, Chapter 6.2: “Induction of hairy roots for symbiotic gene expression studies” [63] using *Agrobacterium rhizogenes*, strain Arqua1. After 14 days of growth on a medium intended for hairy roots emergence (HRE), plants were inoculated with *M. loti* strain MAFF303099 and transferred to pots filled with perlite. After 20 and 54 days, *GUS* activity was analyzed by incubating plants with β-glucuronidase substrate—X-Gluc (Thermo Fisher Scientific, Waltham, MA, USA) dissolved in a standard staining solution, prepared according to the manufacturer instruction. Plants were infiltrated with the substrate under vacuum (−0.07 MPa) for 20 min and then incubated in 37 °C for 3–24 h. Demineralized water was used to stop the enzymatic reaction. Next, transformed roots were mounted in lactic acid and examined using bright field or Nomarsky contrast using Provis AX (Olympus Corporation, Tokyo, Japan) light microscope. Digital images of 3384 × 2708 pixel resolution were saved as tiff files by using a UC90 camera (Olympus Corporation, Tokyo, Japan) under the control of CellSense (SIS) software. The actual color rendition was ensured by specifying the white point outside the section. Further image processing was done using Adobe Photoshop CS6 (Adobe Systems Inc., San Jose, CA, USA), applying the Automatize/Photomerge or Auto-Blend/Panorama tools to assemble single frames into larger images, and the Levels or Curves tool to improve contrast and/or brightness. All adjustments were performed on the entire surface of the image. In individual cases, the image stacking method was used to create an image of structures that could not be properly saved in a single focus. Photo documentation of the GUS activity location was prepared using CorelDraw X5 software (Corel Corporation, Ottawa, ON, Canada).

### 4.7. Microscopic Analysis of Mitotic Divisions

For microscopic observation of mitoses, nodules excised from the oldest part of the root system (at 20 dpi or 54 dpi) were fixed in formaldehyde—acetic acid—ethanol (FAA) [64] overnight, rinsed with 70% ethanol until the odor of acetic acid was not perceivable, and stored in 70% ethanol until further procedure. Next, nodules were rinsed with demineralized water, half-cut to allow better penetration of solutes, and placed in a drop of non-diluted *Aspergillus niger* pectinase (Sigma-Aldrich, St. Louis, MO, USA). After 20–24 h of digestion, nodules were carefully rinsed with demineralized water and placed into a small drop of water on a subject microscope slide. The nodule halves were parted and cells were dislodged using dissecting needles. Although cell clusters were carefully pressed to separate cells, some fragments of nodule endodermis or vascular bundles remained intact. Next, an equal amount of non-diluted glycerol was admixed (at this point, a few microliters of acetocarmine [64] may be added for better visualization of the cells, but no heating should follow), and a cover slide was placed onto the suspension. The cover slides were selected of such a size (20 mm × 20 mm) that the suspension did not flow beyond their edge, ensuring examination of all the nodule cells. Observations were done under Nomarsky contrast using the above described microscope equipment, documentation procedure, and similar image processing.

## Figures and Tables

**Table 1:** *L. japonicus* PINs and their closest *A. thaliana* and *M. truncatula* orthologs, identified using BLAST search.

*L. japonicus* Protein/Gene	*A. thaliana* Orthologous Sequences Identified Using BLAST Search	*M. truncatula* Orthologous Sequences Identified Using BLAST Search
**LjPIN1** (Lj4g3v3114900.1, Lj2g3v0661480.1)	AtPIN1 (At1g73590)	MtPIN4 (MTR_6g069510)
**LjPIN2** (Lj4g3v2139970.1)	AtPIN2 (At5g57090)	MtPIN2 (MTR_4g127100)
**LjPIN3** (Lj0g3v0320849.2)	AtPIN3 (At1g70940)	MtPIN3 (MTR_1g030890)
**LjPIN4** (Lj4g3v0633470.1)	AtPIN3 (At1g70940)	MtPIN1 (MTR_4g084870)
**LjPIN5** (Lj1g3v2809230.1)	AtPIN5 (At5g16530)	MtPIN9 (MTR_7g079720)
**LjPIN6**: LjPIN6a (Lj0g3v0178829.1), LjPIN6b (Lj1g3v0264160.1)	AtPIN6 (At1g77110)	MtPIN6 (MTR_1g029190)
**LjPIN7** (Lj1g3v4106960.1)	AtPIN1 (At1g73590)	MtPIN10 (MTR_7g089360)
**LjPIN8** (Lj2g3v1034600.1)	AtPIN8 (At5g15100)	MtPIN11 (MTR_6g011400)

**Table 2:** Spatial expression pattern of *LjPIN* genes in relation to the root nodule developmental stage. “+,” signal; “−”, no signal; “w,” signal weak; “s,” signal sectorial.

Nodule Developmental Stage		Expression Pattern of *LjPIN* Genes							
		*LjPIN1*	*LjPIN2*	*LjPIN3*	*LjPIN4*	*LjPIN5*	*LjPIN6*	*LjPIN7*	*LjPIN8*
Active initial primordia		−	+	+	+	w	+	+	+
“Hidden” primordia (tissues not differentiated)		+	+	+	+	−	+	+	w
“Emerged” primordia (tissues discernible, nondifferentiated)	Vascular bundle	+	+	−	+	+	+	+	w
	Cortex	−	+	s	+	−	+	−	w
	Vascular bundle–stele connection	+	+	+	+	+	+	+	+
Nodules 20 dpi (young, tissues differentiated)	Vascular bundle	+	−	−	s	+	+	s	−
	Cortex	−	−	−	−	−	−	−	−
	Vascular bundle–stele connection	+	−	+	+	+	+	+	+
Nodules 54 dpi (mature)	Vascular bundle	w	ws	−	w	−	s	−	−
	Cortex	−	−	−	−	−	−	−	−
	Vascular bundle–stele connection	w	w	−	w	+	+	−	+

**Table 3:** Comparison of the location of PIN proteins in particular parts of the *A. thaliana* root tips (based on [40,41,42]) with the expression pattern of the *L. japonicus PIN* promoters observed in this work. Transporters or genes constituting a functional group in a particular species are included in brackets.

Species	Parts of the Root Tip				
	Procambium	Columella Initials-Procambial Initials Boundary	Periblem	Columella	Lateral Root Cap/Protoderm
*A. thaliana*	*AtPIN1, (AtPIN3, AtPIN4, AtPIN7)*	*AtPIN1, (AtPIN3, AtPIN4, AtPIN7)*	*AtPIN1, AtPIN2*	*AtPIN1, (AtPIN3, AtPIN4, AtPIN7)*	*AtPIN1, AtPIN2*
*J. japonicus*	*LjPIN1, LjPIN2, LjPIN3, LjPIN6*	*LjPIN2, LjPIN3, LjPIN6*	*LjPIN2, LjPIN3, LjPIN6*	*LjPIN1, LjPIN2, LjPIN6*	*LjPIN2, (LjPIN3, LjPIN4), LjPIN5, LjPIN6*

## Supplementary Materials

Supplementary materials can be found at http://www.mdpi.com/1422-0067/20/2/235/s1.

## Abbreviations

ABC	ATP-binding cassette
BLAST	Basic Local Alignment Search Tool
bp	base pairs
CDK	cyclin-dependent kinases
CDKB	class B of cyclin-dependent kinases
cDNA	complementary deoxyribonucleic acid
CDS	coding sequence
cll	columella
crh	curled root hair
cv.	cultivar
CYC1	cyclin 1
DNA	deoxyribonucleic acid
DNase	deoxyribonuclease
dpi	day/days postrhizobial inoculation
ER	endoplasmic reticulum
GUS	β-glucuronidase
IAA	indole-3-acetic acid
lc	lateral root cap
LECA	lightweight expanded clay aggregate
NCBI	National Center for Biotechnology Information
nc	nodule cortex
np	nodule primordium
nvb	nodule’s vascular bundle
OD	optical density
p	probability value
PAT	polar auxin transport
pc	procambium
PCR	polymerase chain reaction
PEO-IAA	α-(phenyl ethyl-2-one)-indole-3-acetic acid
PIN	PIN-formed protein
PPFD	photosynthetic photon flux density
prc	meristematic primary root cortex
qPCR	quantitive polymerase chain reaction
RAM	root apical meristem
rc	root primary cortex
RNA	ribonucleic acid
RT-PCR	reverse transcriptase polymerase chain reaction
SE	standard error
v.	version
vs.	versus
*w*/*v*	weight per volume
