# Supplementary material for: Expression Analysis of PIN Genes in Root Tips and Nodules of Lotus japonicus
Source: Int J Mol Sci. 2019 Jan 9;20(2):235. doi: 10.3390/ijms20020235 (PMC6359356; doi:10.3390/ijms20020235)
Supplement: Supplementary file 1 [file ijms-20-00235-s001.pdf]

Article

Expression analysis of *PIN* genes in root tips and nodules of *Lotus japonicus*

Izabela Sańko-Sawczenko [1], Dominika Dmitruk [1], Barbara Łotocka [1], Elżbieta Różańska [1] and Weronika Czarnocka [1,*]

[1] Department of Botany, Faculty of Agriculture and Biology, Warsaw University of Life Sciences, Nowoursynowska 159, 02-776 Warsaw, Poland; izabela_sanko_sawczenko@sggw.pl (I.S-S.); dominika_dmitruk@sggw.pl (D.D.); barbara_lotocka@sggw.pl (B.Ł.); elzbieta_rozanska@sggw.pl (E.R.)

* Correspondence: weronika_czarnocka@sggw.pl; Tel.: +48-22-59-326-6

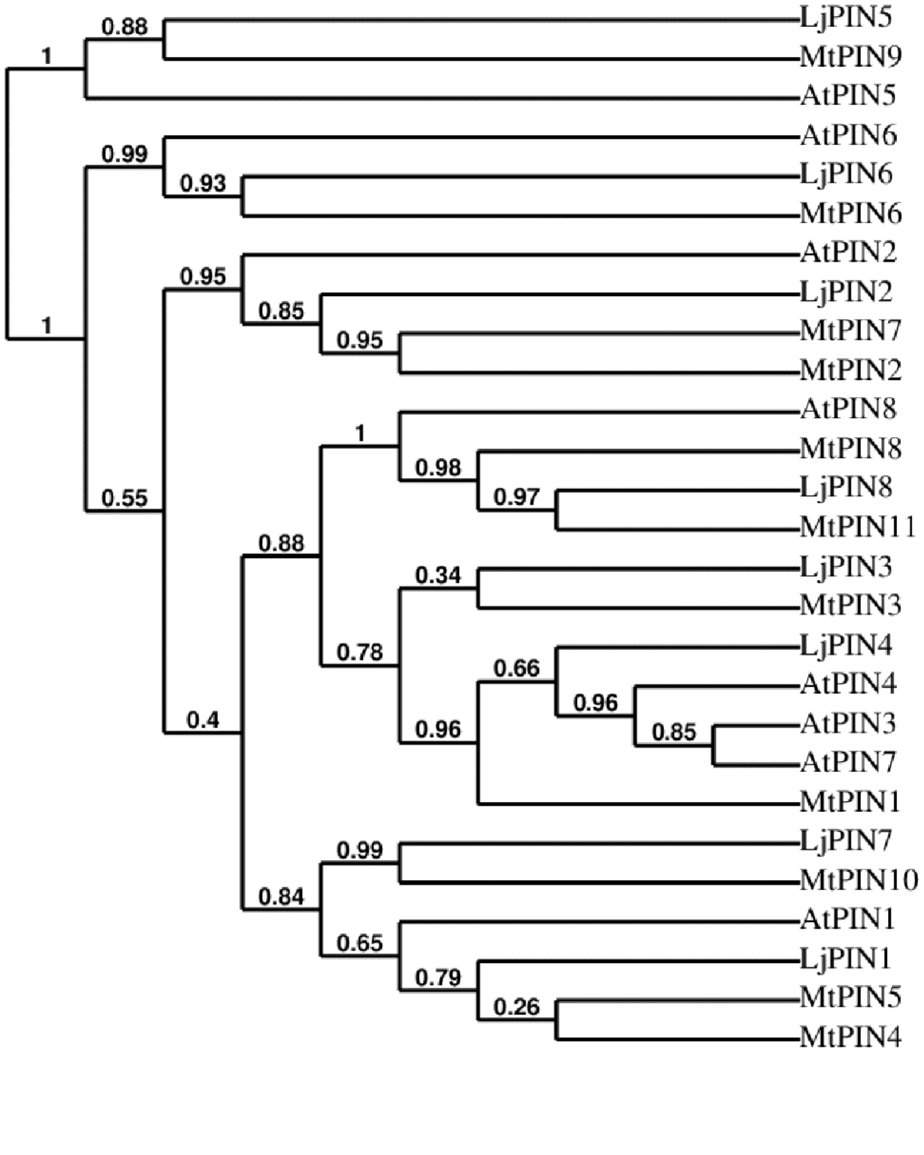

Figure S1. Cladogram presenting the phylogenetic relationship of PIN proteins from *L. japonicus* (Lj), *M. truncatula* (Mt) and *A. thaliana* (At).

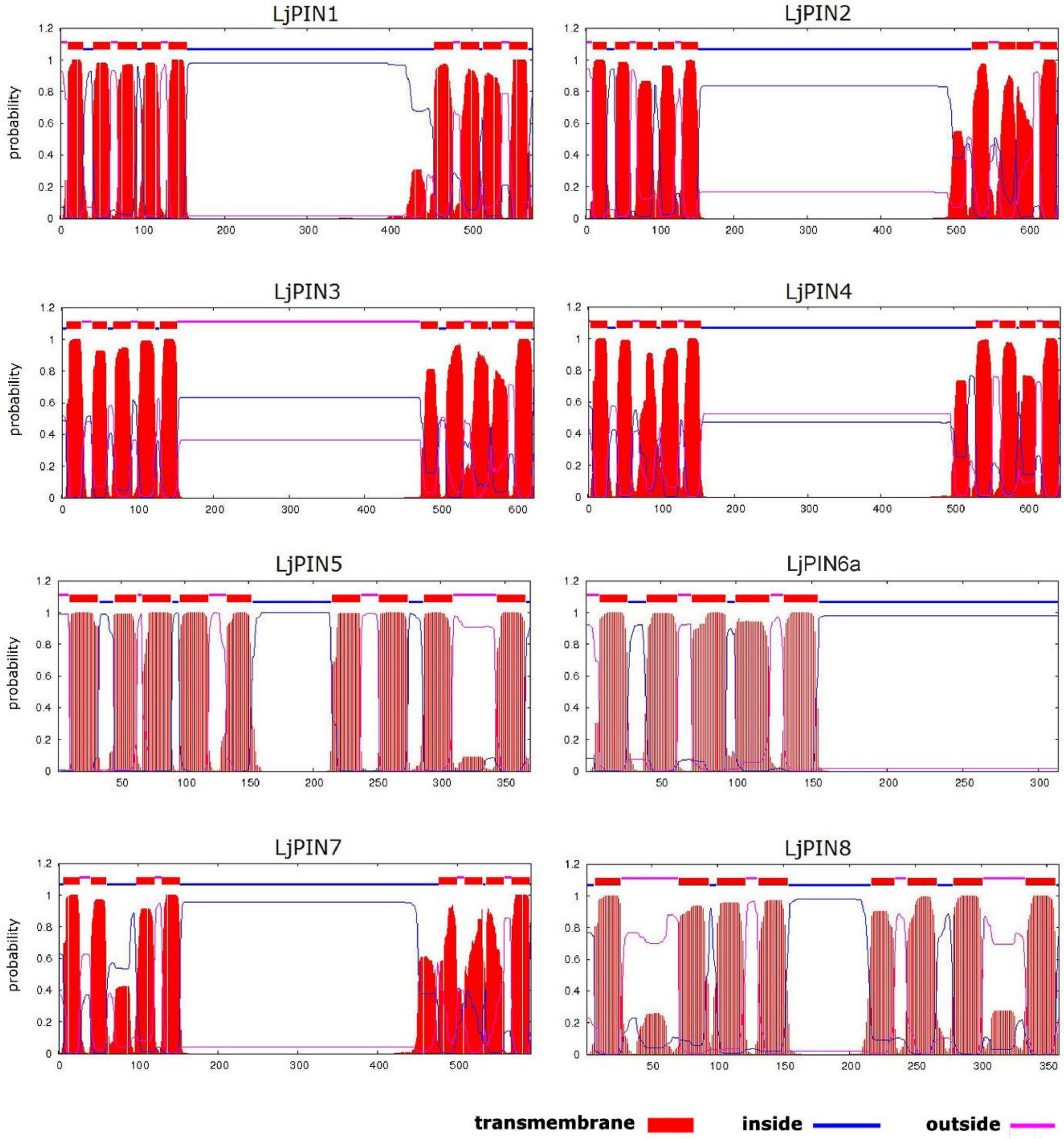

Figure S2. Transmembrane topology of *L. japonicus* PIN proteins. Red peaks represent transmembrane helices.

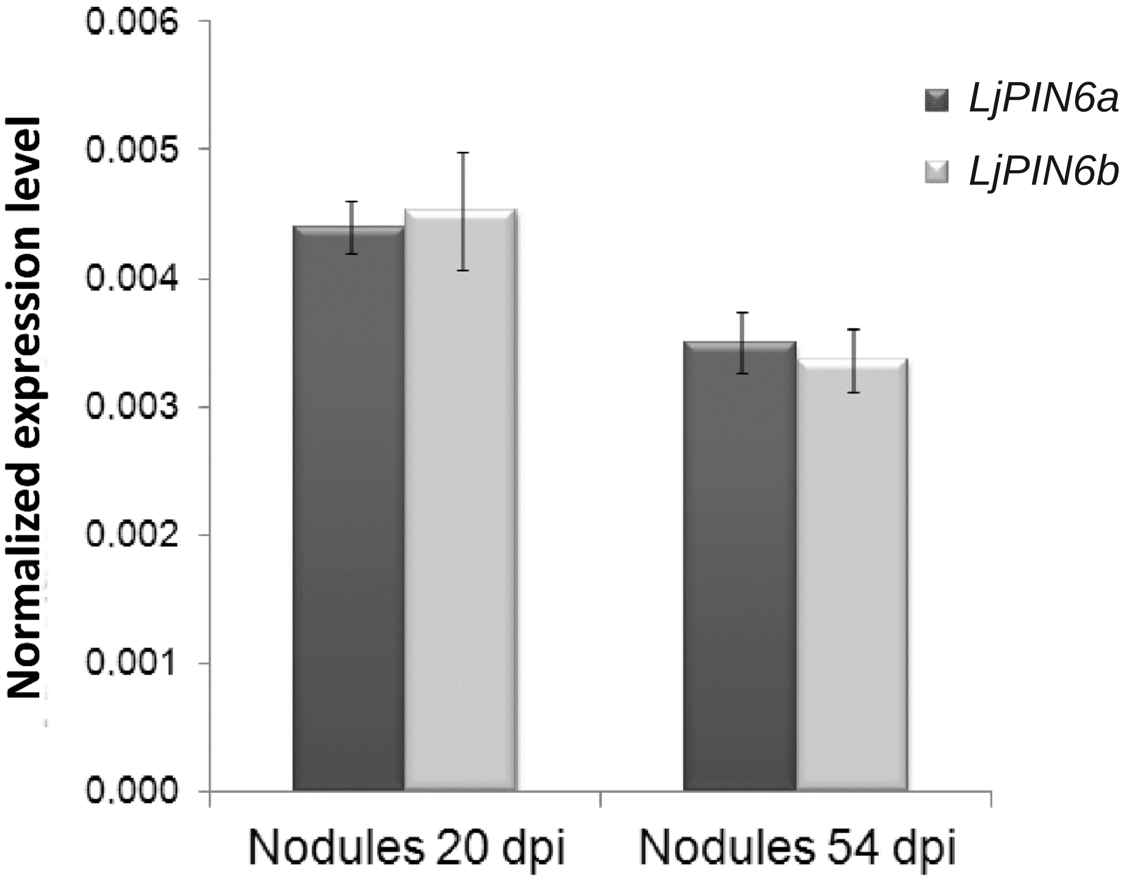

Figure S3. Normalized expression level of *LjPIN6a* and *LjPIN6b* in root nodules. Mean values (±SE) are derived from three biological replicates, for which three individual qPCR reactions were performed.

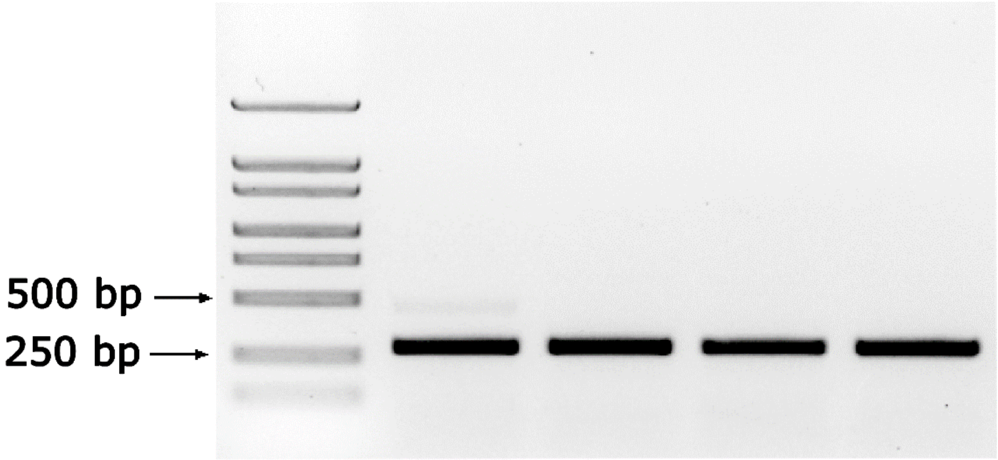

Figure S4. The electrophoretic separation of LjPIN6 fragment amplification products. bp- base pairs.

A

LjPIN6 – complete coding sequence (CDS)

ATGGTTACAGGAGATGATTTGTACAACGTGATGTGCGCCATGGTGCC
TCTATATTTCGCAATGCTGGTAGCTTACGCATCAGTGAAATGGTGCA
AAATGTTCACCCCACAGCAGTGCTCCGGCGTCAACCGTTTCGTGGCG
GTGTTTGCGATTCCGGTTCTCTCTTTCCATTTCATTTCCATCAACAATC
CTTATGAGATGGACGCTAAGTTCATCATAGCGGACACGCTCTCCAAG
CTCGTCGTGCTGTTCTTCCTCTTTCTCTGGGCTATTTTTTTTGCTTCTG
GATCTCTCGACTGGCTTATCACGCTCTTCTCGCTCGCAACCTTGCCCA
ACACGCTTGTCATGGGGATTCCTCTACTCCAAGCCATGTACGGCCAA
TTCACGCAAGGCCTCATGGTGCAACTCGTTGTACTCCAATGCATCATA
TGGTATACTCTACTGTTGTTTCTATTGGAATACAGAGCAGCGACCCTT
TTGATCAAAACCGAGTTCCCTGGGGACACGGCGGCGTCCATAACCAA
AATCGAGGTCGACGGCGACGTGATTTCCCTCGACGGCCACGACGTGC
CACTGCAAACGCAGTCGGAAACCGACACCCACGGCCGCATCAGCGT
GCGAATCCGGCGATCAATCTCCTCAGCTCCGGACTCAACCTCATCAA
TCGGCAACGCCGTTATCACTCCAAGGCATTCGAATCTCACCAACGCC
GAGCACCTGCTCGACGGCGACCCCTCCTTTGGATACCCGCCGGCGAG
CCCGAGGTTATCCGGATGCGCCTCCTCCGACGCGTACTCGCTTCAGCC
CACGCCGCGGGCGTCAAATTTCAATGAGACGGAGGTCACGGCGGGA
ACGCCGGTATGGGGGAGGTCTCCGGTGGGCGGCGGGAGGGTTTCTCG
GCAGATGTCGCCGGGGAAATGCCAAGTAGAAGAGAGACAG**GGATGC**
AAGGACATTACGCAGTCAGATAAAGAAATTAGCTTCAGAGACAAC
ATCAAAGTTTCAATGCCAGGGGAAGAAGCTGCAGACACAACTGCTC
GTAACCAGAAAATGCCACATGCTTTTGTCATGATAAGGCTTATACTT
ACAGTAGTGGGAAGAAAACTTTCGCGTAACCCTAATACATATTCTAG
TGTATTAGGACTTGTTTGGTCTCTAATCTCCTTCAAATGGAACATGGA
AATGCCTAGTCTGATCAAAGCATCTATCAAAATCATCTCGGATGCAA
GCCTTGGAATGGCTATGTTTAGCTTAGGGCTTTTCATGGCCCTTCAGC
CTCGTATCATTGCTTGTGGTACCAAAAGAGCAGCCATGGGAATGGCC
ATTCGTTTCGTGTGCGGGCCTCTTGTAATGTCAGCATCCTCCATTGTC
ATTGGATTGAGAGGGGACAAATTGCACACAGCAATTGTACAGGCTGC
ACTTCCACAGGGGATTGTACCATTTGTATTTGCAAGGGAATATGGGC
TGCATCCTGATATTTTGAGCACAGGGGTTATCTTTGGCATGCTAGTAT
CCTTACCAATAACTCTCCTATATTACATACTTCTTGGCTTGTGA

LjPIN6 – complete protein sequence

MVTGDDLYNVMCAMVPLYFAMLVAYASVKWCKMFTPQQCSGVNRFV
AVFAIPVLSFHFISINNPYEMDAKFIIADTLSKLVVLFFLFLWAIFFASGSL
DWLITLFSLATLPNTLVMGIPLLQAMYGQFTQGLMVQLVVLQCIIWYTL
LLFLLEYRAATLLIKTEFPGDTAASITKIEVDGDVISLDGHDVPLQTQSET
DTHGRISVRIRRSISSAPDSTSSIGNAVITPRHSNLTNAEHLLDGDPSFGYP
PASPRLSGCASSDAYSLQPTPRASNFNETEVTAGTPVWGRSPVGGGRVS
RQMSPGKCQVEERQGCKDITQSDKEISFRDNIKVSMPGEEAADTTARNQ
KMPHAFVMIRLILTVVGRKLSRNPNTYSSVLGLVWSLISFKWNMEMPSL
IKASIKIISDASLGMAMFSLGLFMALQPRIIACGTKRAAMGMAIRFVCGP
LVMSASSIVIGLRGDKLHTAIVQAALPQGIVPFVFAREYGLHPDILSTGVI
FGMLVSLPITLLYYILLGL-

B

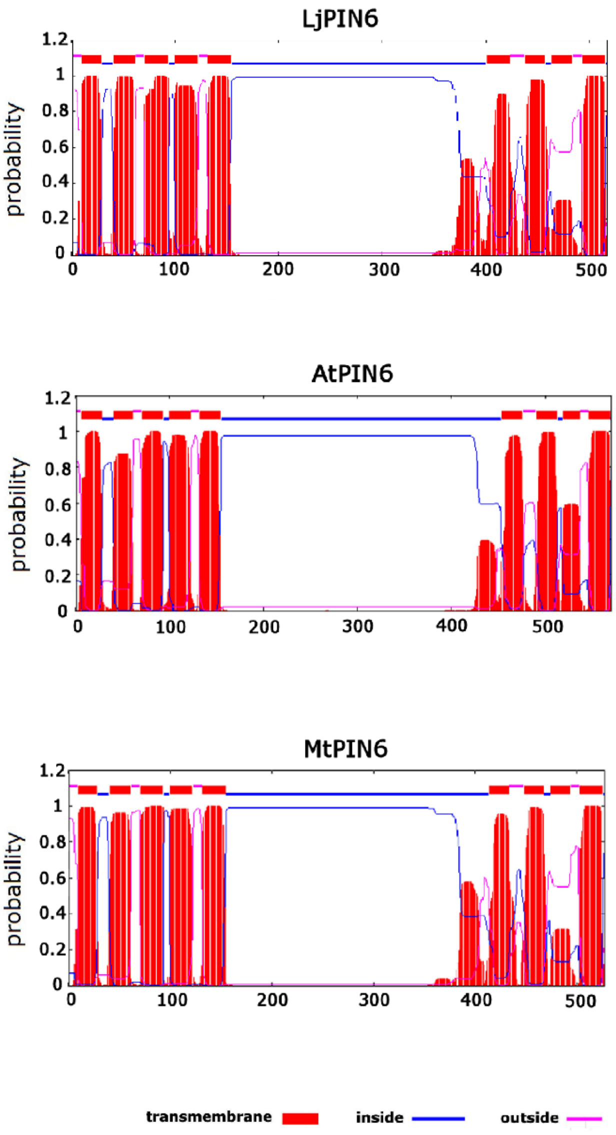

Figure S5. Complete sequence of LjPIN6 and its transmembrane domain topology. (**A**) LjPIN6 coding and protein sequence with the fragment obtained through the sequencing (bolded) and binding sites of primers used for sequencing (shaded). (**B**) Transmembrane domain topology of LjPIN6 and its orthologs from *A. thaliana* and *M. truncatula*. Red peaks represent transmembrane helices.

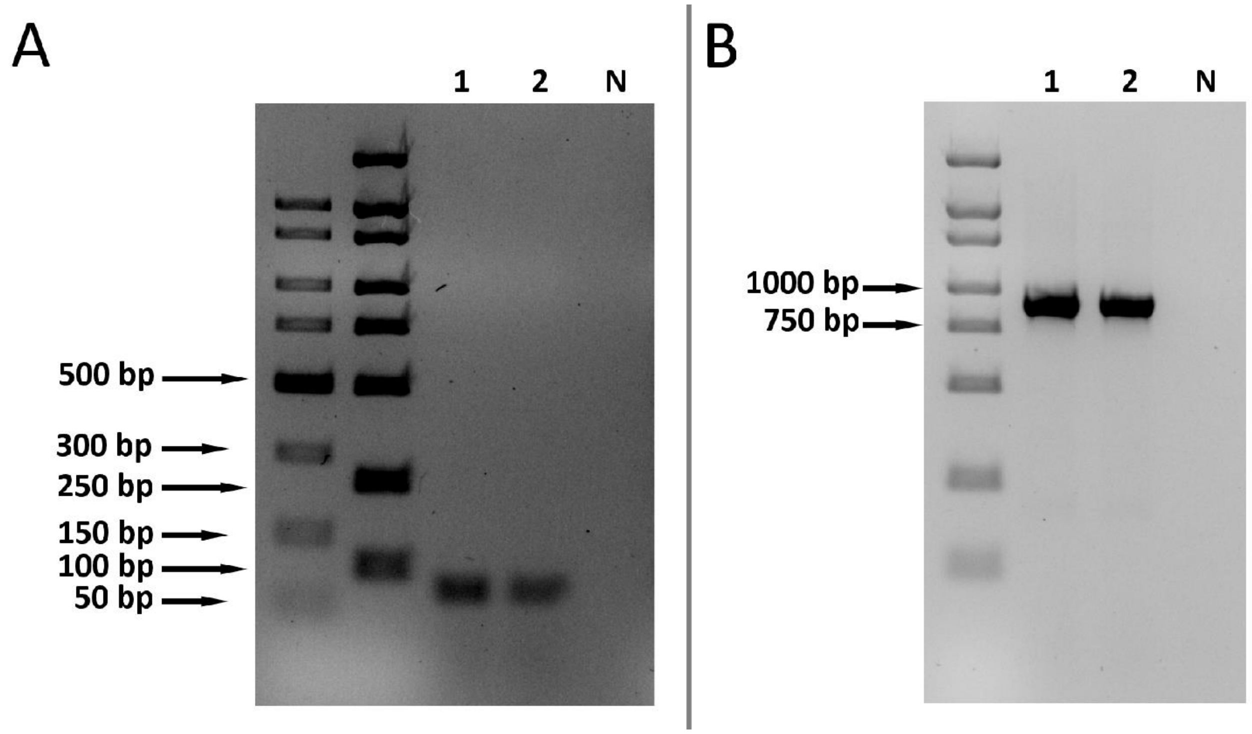

Figure S6. The electrophoretic separation of *LjPIN5* (**A**) and *LjPIN8* (**B**) products of amplification performed on genomic *L. japonicus* DNA template. Wells number 1 and 2 represent two biological repetitions, wells "N" state for negative controls (PCR performed in the same conditions as other samples, with the same volume of ingredients, but without DNA template). bp- base pairs.

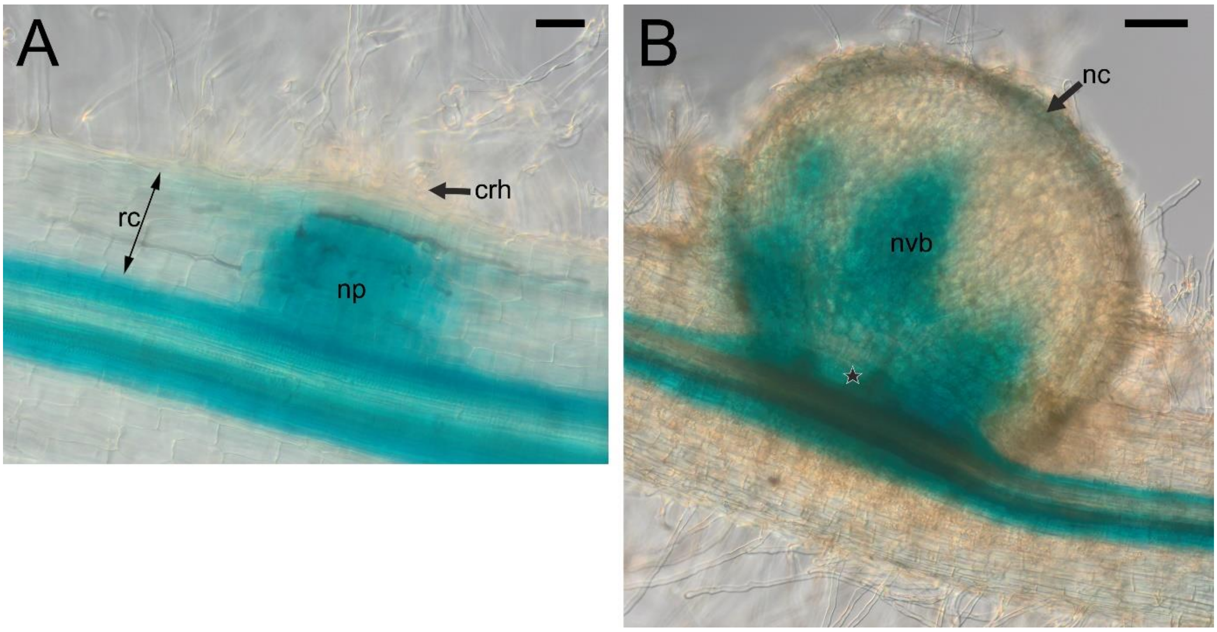

Figure S7. The pattern of *LjPIN1* expression in the "hidden" (**A**) and "emerged" (**B**) root nodule primordia. Labels: crh – curled root hair; nc – nodule cortex; np – nodule primordium; nvb – nodule vascular bundle; rc – root primary cortex; asterisk – vascular connection with the root stele within the nodule base. Bars: 50 μm (A), 100 μm (B).

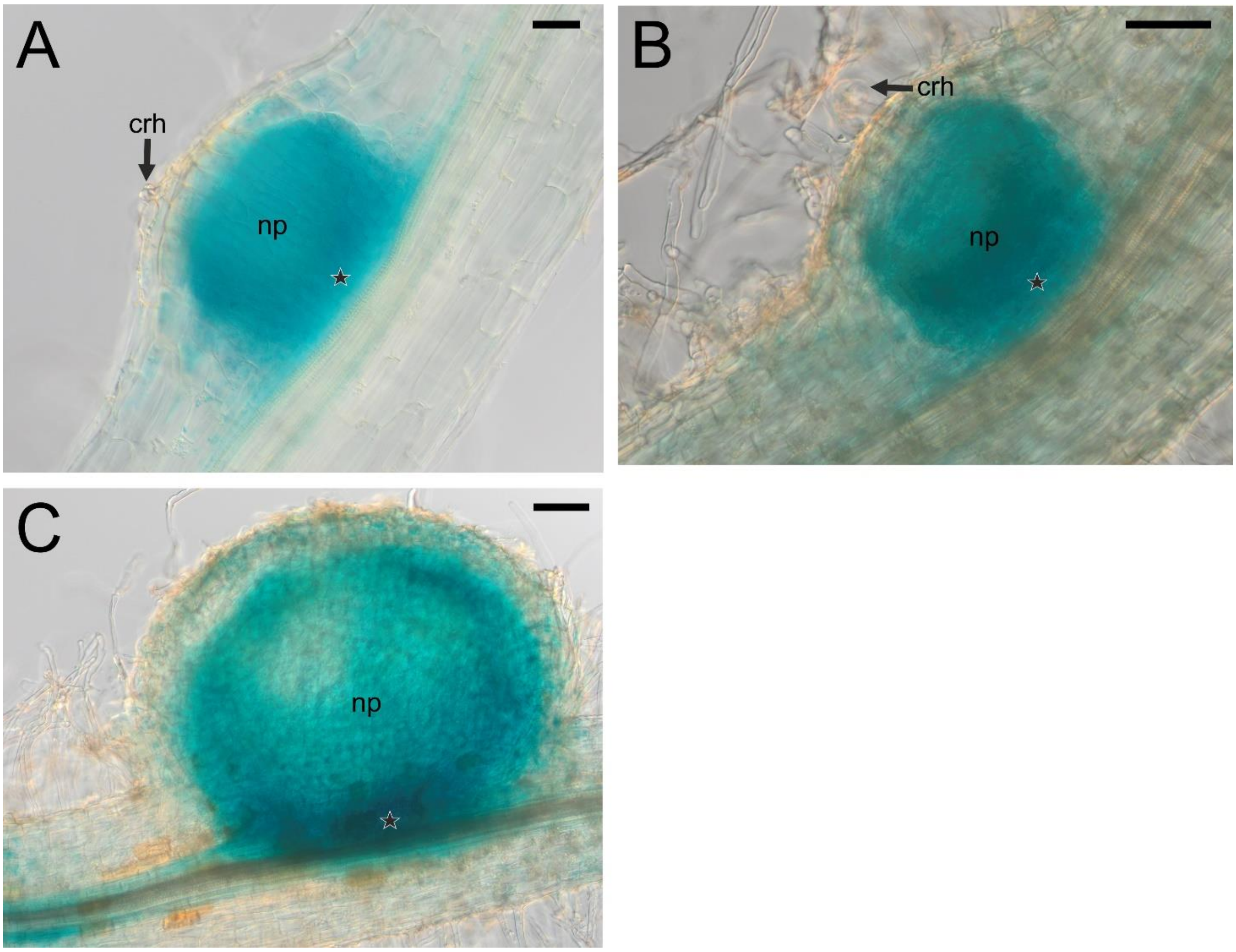

Figure S8. The pattern of *LjPIN2* expression in the "hidden" (**A**, **B**) and "emerged" (**C**) root nodule primordia. Labels: crh – curled root hair; np – nodule primordium; nvb – nodule vascular bundle; asterisk – vascular connection with the root stele within the nodule base. Bars: 50 μm (A), 100 μm (B, C).

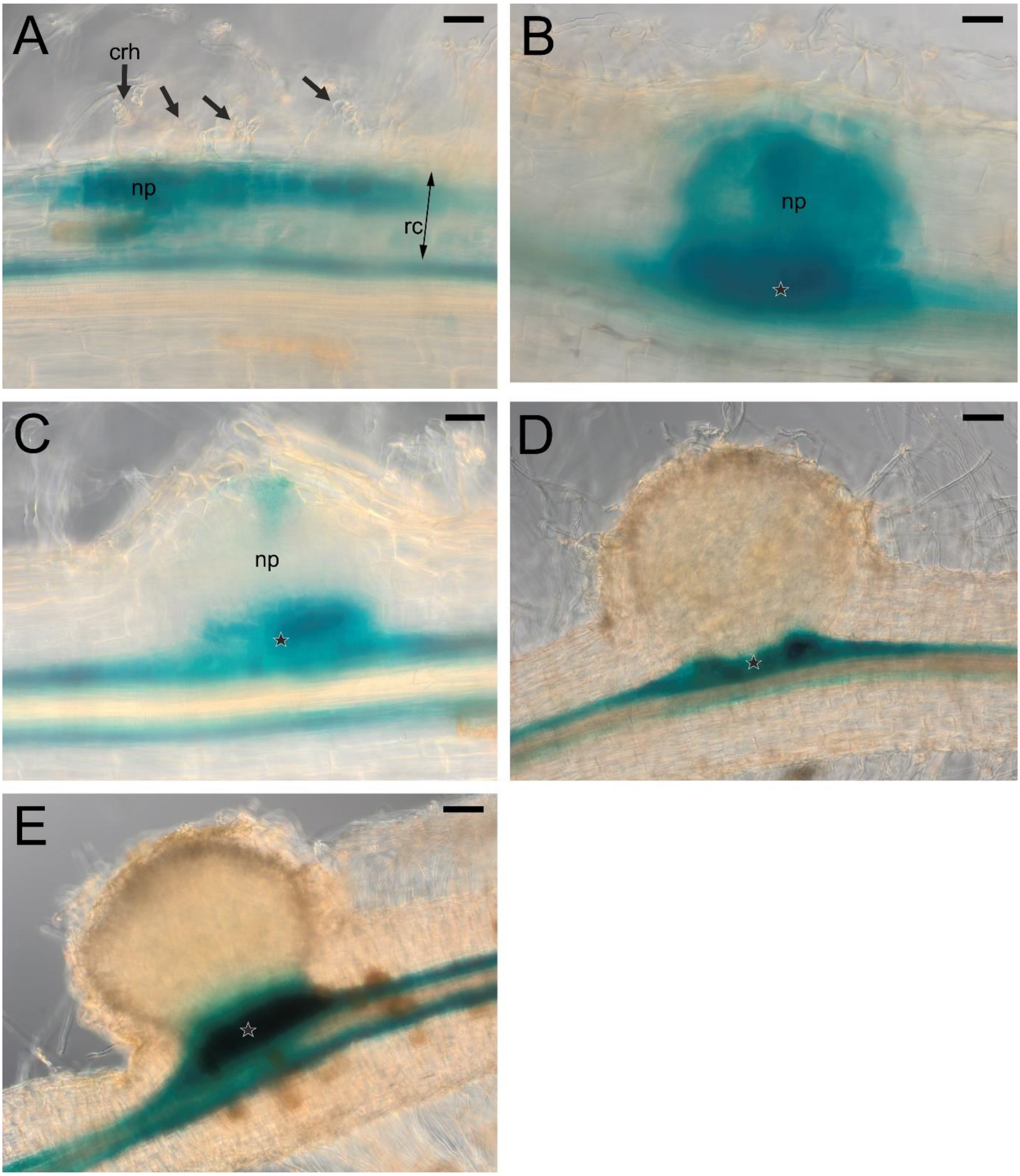

Figure S9. The pattern of *LjPIN3* expression in the initial (**A**), "hidden" (**B**), "emerging" (**C**) and “emerged” (**D**) root nodule primordia and in juvenile, 20 dpi, nodules (**E**).
Labels: crh – curled root hair; np – nodule primordium; rc – root primary cortex; asterisk – vascular connection with the root stele within the nodule base. Bars: 50 μm (A-C), 100 μm (D, E).

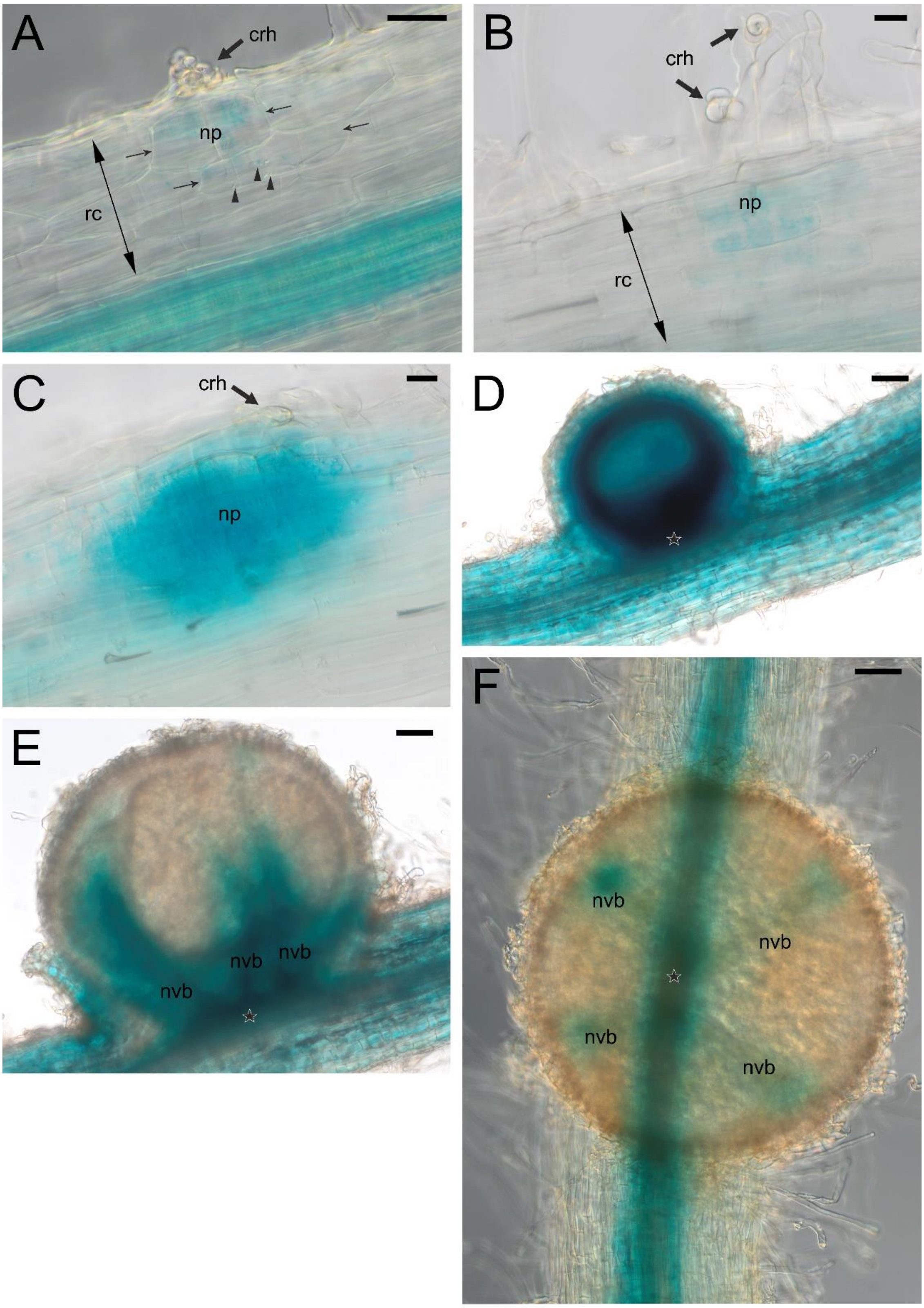

Figure S10. The pattern of *LjPIN4* expression in the initial (**A**, **B**, **C**) and "emerged" (**D**) root nodule primordia and in juvenile, 20 dpi, nodules (**E**, **F**).

Labels: crh – curled root hair; np – nodule primordium; nvb – nodule vascular bundle; rc – root primary cortex; thin black arrows – divided cells of root cortex constituting initial primordium of the nodule; arrowheads – starch grains; asterisk – vascular connection with the root stele within the nodule base. Bars: 20 μm (B, C), 50 μm (A), 100 μm (D-F).

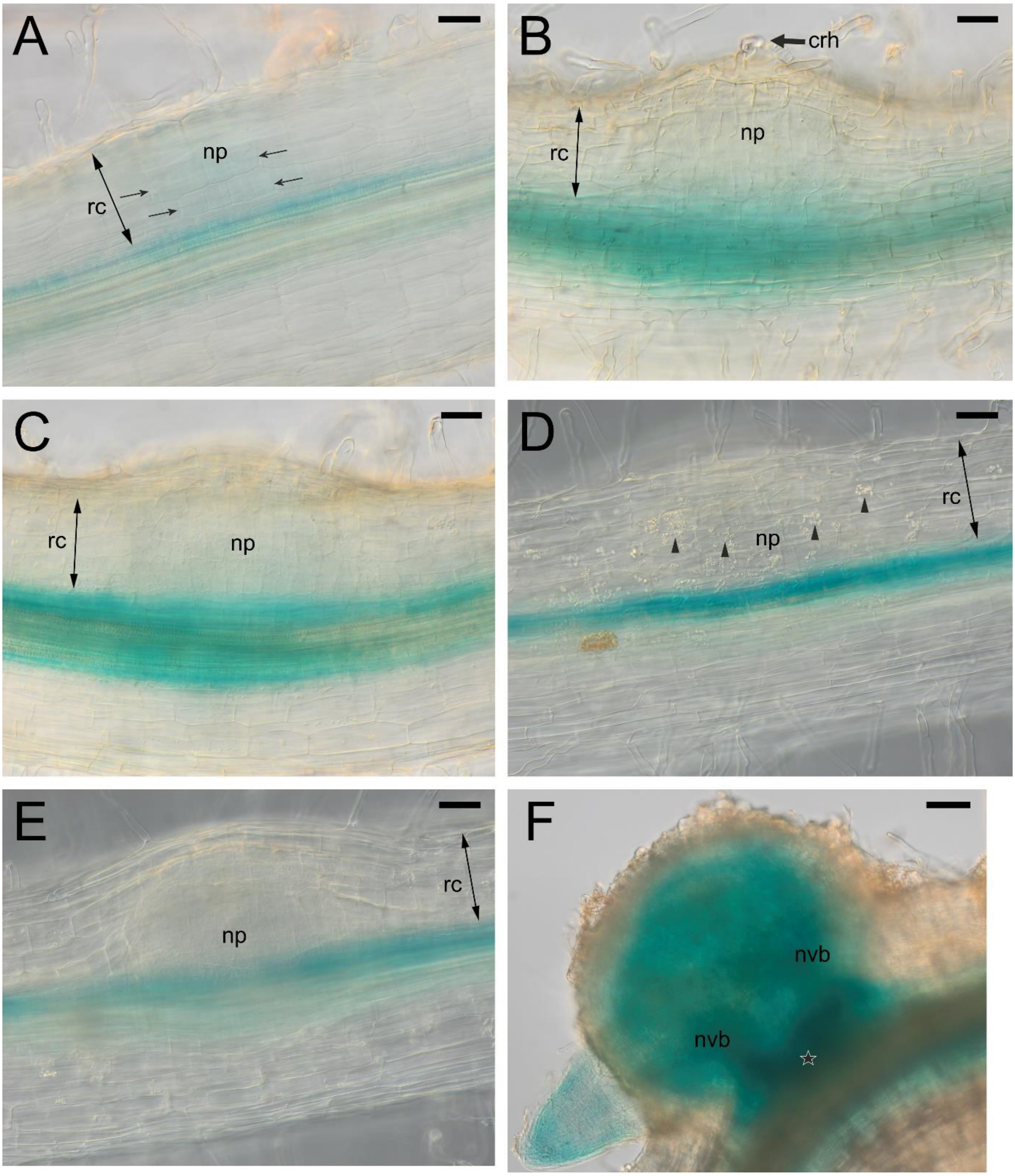

Figure S11. The pattern of *LjPIN5* expression in the initial (**A**), “hidden” (**B**, **C**, **D**, **E**) and “emerged” (**F**) root nodule primordia.

Labels: crh – curled root hair; np – nodule primordium; nvb – nodule vascular bundle; rc – root primary cortex; thin black arrows – divided cells of root cortex constituting initial primordium of the nodule; arrowheads – starch grains; asterisk – vascular connection with the root stele within the nodule base. Bars: 50 µm (A-C), 100 µm (D-F).

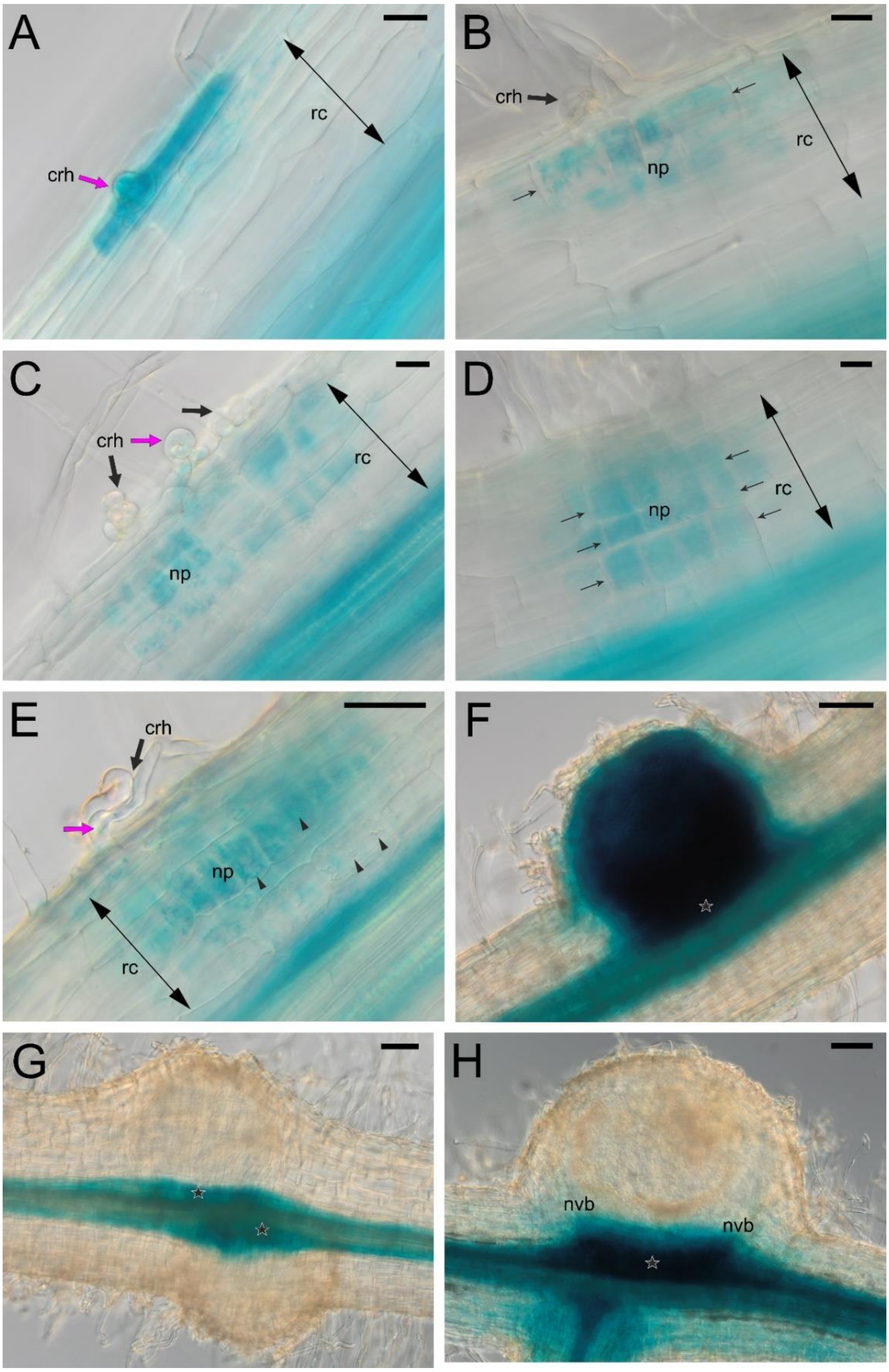

Figure S12. The pattern of *LjPIN6* expression in the initial (**A**, **B**, **C**, **D**, **E**) and "emerged" (**F**, **G**) root nodule primordia and in juvenile, 20 dpi, nodules (**H**).

Labels: crh – curled root hair; np – nodule primordium; nvb – nodule vascular bundle; rc – root primary cortex; pink arrow – curled root hair expressing GUS signal; thin black arrows – divided cells of root cortex constituting initial primordium of the nodule; arrowheads – starch grains; asterisk – vascular connection with the root stele within the nodule base. Bars: 20 µm (A-D), 50 µm (E), 100 µm (F-H).

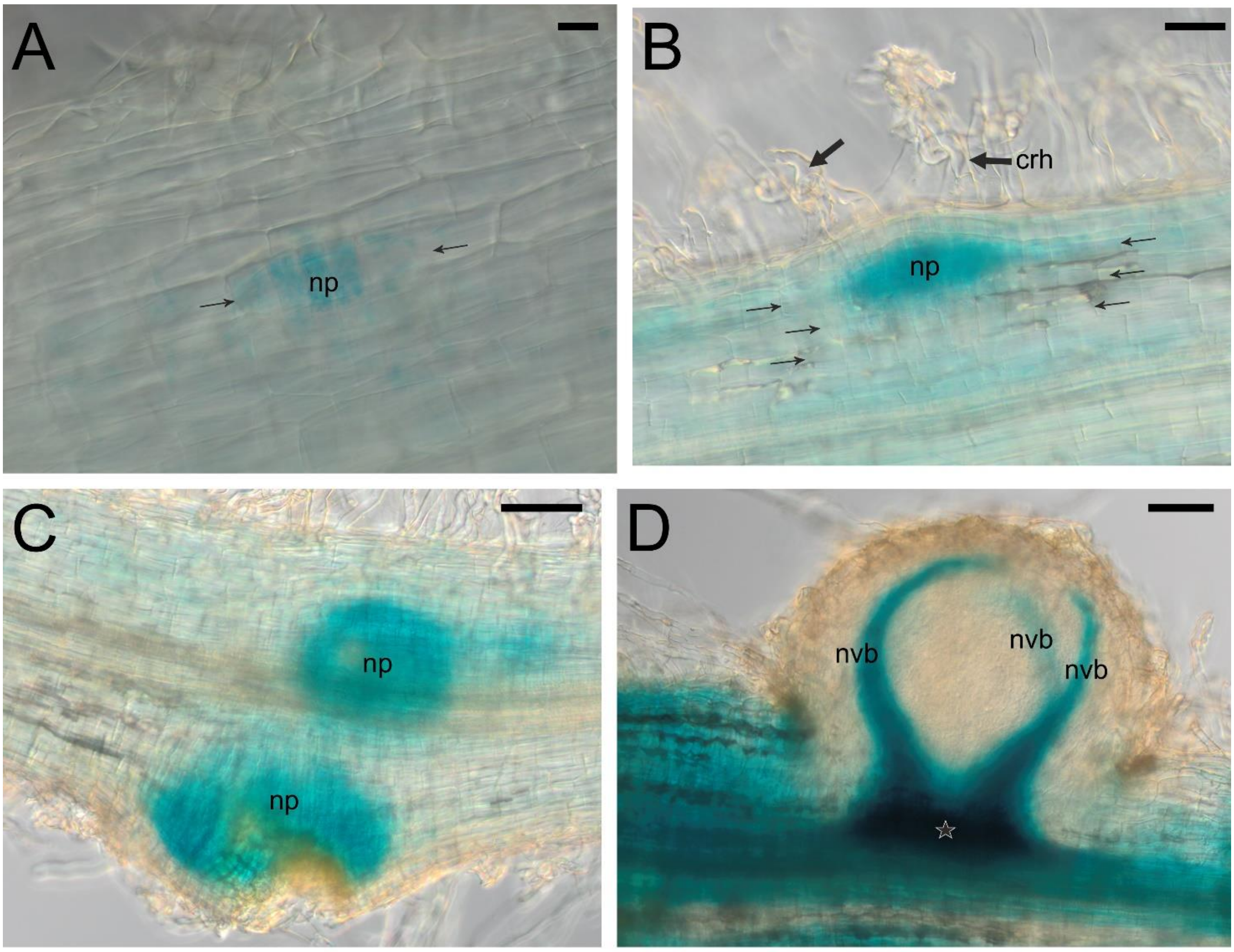

Figure S13. The pattern of *LjPIN7* expression in the initial (**A**, **B**), "hidden" (**C**) and "emerged" (**D**) root nodule primordia and in juvenile, 20 dpi, nodules (**E**).

Labels: crh – curled root hair; np – nodule primordium; nvb – nodule vascular bundle; thin black arrows – divided cells of root cortex constituting initial primordium of the nodule; asterisk – vascular connection with the root stele within the nodule base. Bars: 20 μm (A), 50 μm (B), 100 μm (C, D).

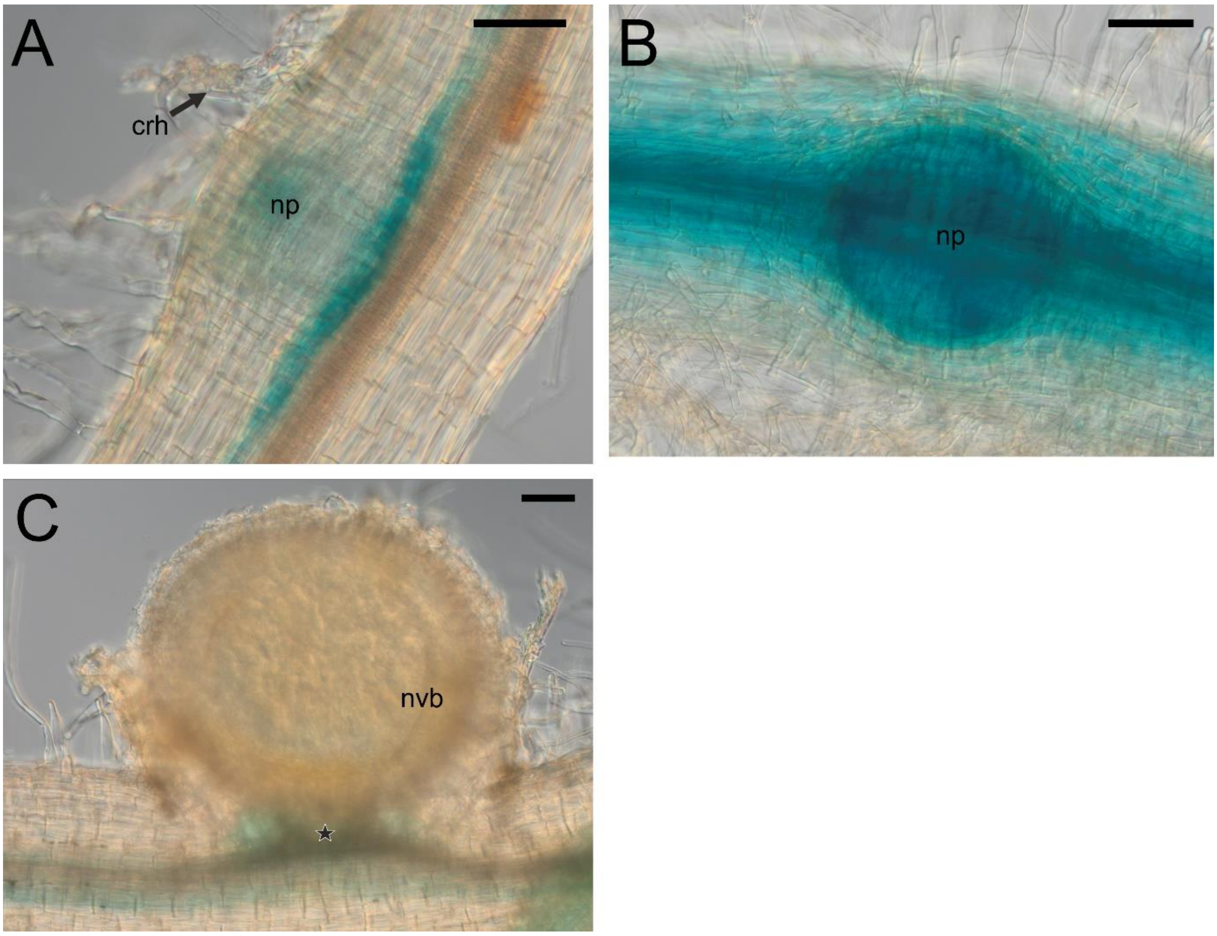

Figure S14. The pattern of *LjPIN8* expression in the initial (**A**) and "hidden" (**B**) root nodule primordia and in juvenile, 20 dpi, nodules (**C**).

Labels: crh – curled root hair; np – nodule primordium; nvb – nodule vascular bundle; asterisk – vascular connection with the root stele within the nodule base. Bars: =100 μm.

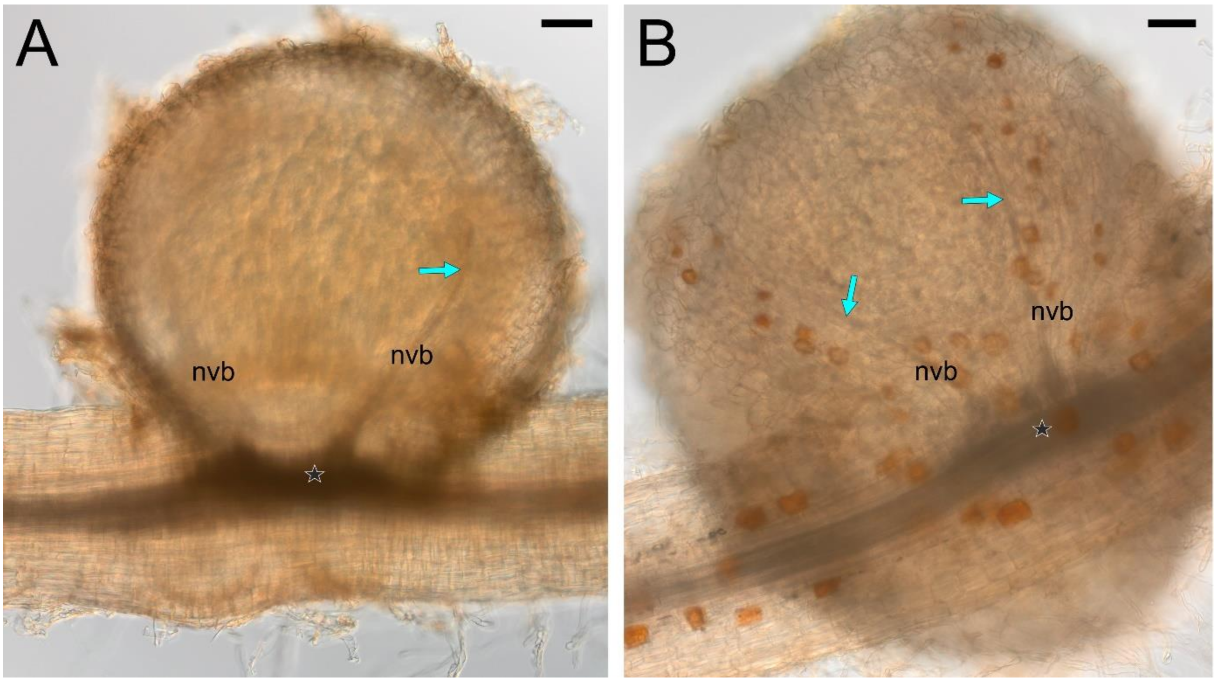

Figure S15. The control *L. japonicus* nodules collected from plants with adventitious roots formed after cutting off the root system (as in regular transformation process) but not transformed with *A. rhizogenes* (**A**), and plants transformed with non-transgenic *A. rhizogenes* (**B**).
Labels: nvb – nodule vascular bundle; blue arrows – files of tracheary elements in the vascular bundle; asterisk – vascular connection with the root stele within the nodule base. Bars: 100 μm.

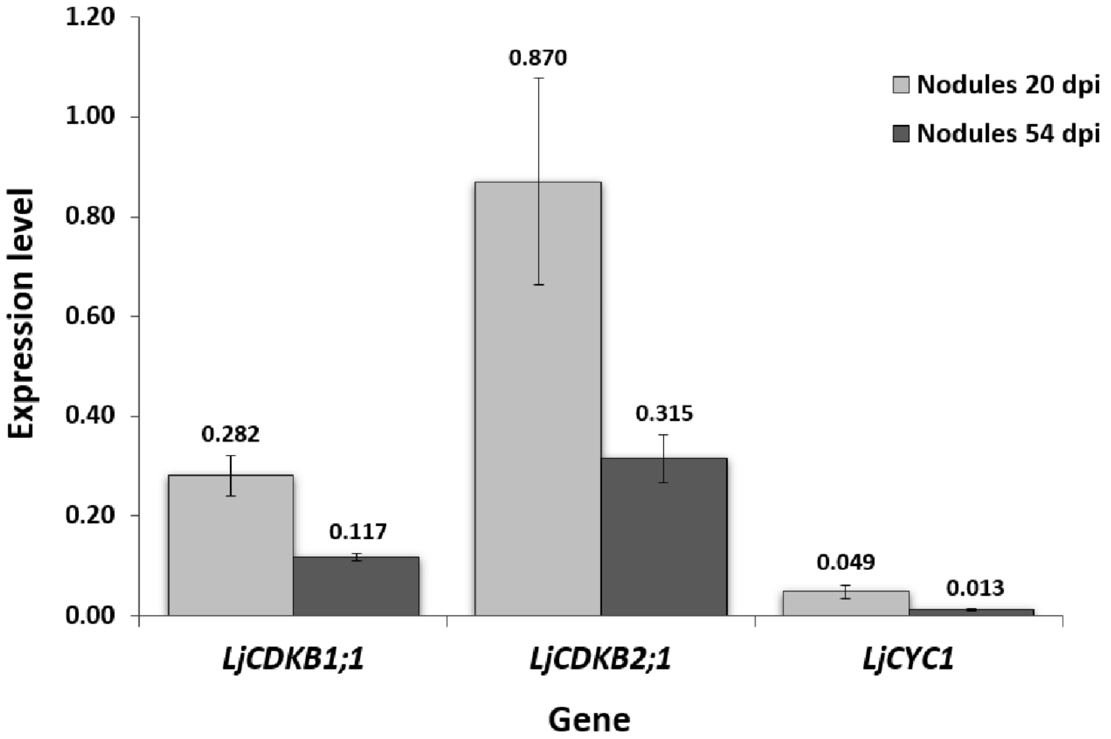

Figure S16. Normalized expression level of G2-M phase cell cycle markers. Mean values (±SE) are derived from three biological replicates, for which three individual qPCR reactions were performed.

Table S1. RT-PCR conditions.

Temperature	Time
98 °C	30 s
35 cycles:	
98 °C	10 s
59.5 °C	30 s
72 °C	1 min
72 °C	10 min

Table S2. Real-time qPCR conditions.

Temperature	Time
PCR	
50 °C	20 s
95 °C	10 min
40 cycles:	
95 °C	15 s
60 °C	1 min
Melting curve	
95 °C	15 s
60 °C	1 min
95 °C	30 s
60 °C	15 s

Table S3. Accessions of genes, sequences of primers and product lengths for real-time qPCR. bp - base pairs.

Gene / ID	Forward primer sequence 5′ → 3′	Reverse primer sequence 5′ → 3′	Product length [bp]
LjPIN1 (Lj4g3v3114900.1)	ATACTCTCTACAATCCTCAAGGAACC	GTTCCACCAGAAATAGCATCATAGT	150
LjPIN2 (Lj4g3v2139970.1)	CCCACTATTGTAAAAGGTTCCATC	AGCCATAGAAAATGTTGCTACAGA	138
LjPIN3 (Lj0g3v0320849.2)	ATCGCTATGTACGGCGATTACT	GGAGTCAACCTTGAATGACACAAT	174
LjPIN4 (Lj4g3v0633470.1)	ATTTCTTACAGGTCCTGCAGTTATG	GGTAGAGCTATCAACATCCCAAATA	192
LjPIN5 (Lj1g3v2809230.1)	TCGCCCTGTTTTTAGGCTAC	TATCGCATCGCACTGTTCTC	71
LjPIN6a (Lj0g3v0178829.1)	CTCTATATTTCGCAATGCTGGTAG	CTATGATGAACTTAGCGTCCATCTC	174
LjPIN6b (Lj1g3v0264160.1)	GAAAACTTTCGCGTAACCCTAATA	CATGAAAAGCCCTAAGCTAAACAT	167
LjPIN7 (Lj1g3v4106960.1)	GCTAGTGTCATGACTAGGCTCATTT	GCCTGCATCAGATAGAATTGATATT	171
LjPIN8 (Lj2g3v1034600.1)	ATAACCGGTCTATCATTGTCAACAC	TGTAGCTCCTGAGAAGTCTCACTTT	242
LjCDKB1;1 (Lj3g3v2061650.1)	CGATCTCAAGAAATACATCGATACC	TACAGAGCTGAAACAGAAAACTCTGAA	95
LjCDKB2;1 (Lj5g3v0279150.1)	AGGAACTTATGGGAAGGTGTATAGG	TGTTTAACATCCATTAACCTGACAA	180
LjCYC1 (Lj2g3v1645400.1)	CATATGTTTTCATGAGAAGGTTCCTA	CATACTCAACTAGAGACAGCTCAACC	99
LjPP2AA1 (Lj2g3v1155670)	GCAGTGCTTATAGACGAGCTGA	TGGATAGCTTGCGAATTGAG	70
LjPP2AA2 (Lj2g3v0742070)	TCGACGAGTTGAAGAACGAG	GTGTCCTCTCCTCACCAAGC	93

Table S4. PCR conditions.

Temperature	Time
95 °C	5 min
40 cycles:	
95 °C	30 s
60 °C	30 s
72 °C	30 s
72 °C	5 min

Table S5. Sequences of primers for promoter amplification. Shaded parts of the sequence represent fragments for Gateway cloning, while underlined nucleotides in the primers for elongation PCR show complementarity to the template.

	**Forward primer sequence 5′ → 3′**	**Reverse primer sequence 5′ → 3′**	**Position of promoters**
pLjPIN1	AAAAAGCAGGCTCCTAAACCATTGTTCCGATCTTAC	AGAAAGCTGGGTCTTCTTCACTTCGGTAGAGAAACA	Chromosome 4 42168645..42170156
pLjPIN2	AAAAAGCAGGCTCCATAGGGAACCTCCTAGATCG	AGAAAGCTGGGTCGGCTAAACCAAGGTTGTTGT	Chromosome 4 (-) 29544633..29546630
pLjPIN3, PCRI	AAAAAGCAGGCTCCTCCCATGAGTCAAGTTATCC	GTACTCAAAGAGGAATAAGAGGAG	-
pLjPIN3, PCRII	AAAAAGCAGGCTCCTCCCATGAGTCAAGTTATCC	AGAAAGCTGGGTCCTTTTTTTTGTTGGGTTTTA	Chromosome 0 (-) 167444230..167446018
pLjPIN4	AAAAAGCAGGCTCCAAGAATAAGCCTCTCGTTTCTCAA	AGAAAGCTGGGTCTTTCTTTATATGGCCAAAAAAAAG	Chromosome 4 10020316..10021906
pLjPIN5, PCRI	AAAAAGCAGGCTCCTATGATGATGTCCAAGCTGA	GAGAAGCAAAGTGAGCCATA	-
pLjPIN5, PCRII	AAAAAGCAGGCTCCTATGATGATGTCCAAGCTGA	AGAAAGCTGGGTCCTTTTTTTAATTTTTTTCTTGTCTCTCG	Chromosome 1 (-) 31944557..31946536
pLjPIN6	AATTCTAAGGTGGGGTGGTTAAAAA	CTTTTGAGTGAGATAGAAAGTGGTGT	Chromosome 1 Unmapped region
pLjPIN7	AAAAAGCAGGCTCCAGAAATTTAAAACCCACAATTCCA	AGAAAGCTGGGTCTTGTTTTAGCAGAAACAATCACT	Chromosome 1 48774196..48776085
pLjPIN8	AAAAAGCAGGCTCCTTCATACAGAATCACCACGA	AGAAAGCTGGGTCGATTCCTAAGTATTGAAAGA	Chromosome 2 (-) 16542704..16544503
Elongation PCR pLjPIN6	GGGGACAAGTTTGTACAAAAAAGCAGGCTCCAATTCTAAGGTGG	GGGGACCACTTTGTACAAGAAAGCTGGGTCTTTTGAGTGAGATA	-
Elongation PCR pLjPIN1-5, pLjPIN7-8	GGGGACAAGTTTGTACAAAAAAGCAGGCT	GGGGACCACTTTGTACAAGAAAGCTGGGT	-

Table S6. Conditions of promoter regions amplification.

Temperature	Time
98 °C	5 min
35 cycles:	
98 °C	30 s
58 °C – *pLjPIN6*<br>60 °C – *pLjPIN: 2*, *3*, *8* (PCRI, PCRII), *5* (PCRII)<br>66 °C – *pLjPIN: 1*, *4*, *7*<br>70 °C – *pLjPIN5* (PCRI)	30 s
72 °C	1 min
72 °C	5 min

Table S7. Conditions of promoter regions elongation with *att* sequences.

Temperature	Time
98 °C	30 s
30 cycles:	
98 °C	10 s
60 °C	30 s
72 °C	1 min
72 °C	5 min
